# The role of the interplay between macrophage glycolytic reprogramming and NLRP3 inflammasome activation in acute lung injury/acute respiratory distress syndrome

**DOI:** 10.1002/ctm2.70098

**Published:** 2024-12-02

**Authors:** Lan Luo, Xiaoli Zhuang, Lin Fu, Ziyuan Dong, Shuyuan Yi, Kan Wang, Yu Jiang, Ju Zhao, Xiaofang Yang, Feilong Hei

**Affiliations:** ^1^ Department of Extracorporeal Circulation and Mechanical Circulation Assistants Center for Cardiac Intensive Care Beijing Anzhen Hospital Capital Medical University Beijing China

**Keywords:** acute lung injury/acute respiratory distress syndrome, glycolytic reprogramming, macrophage, NLRP3 inflammasome

## Abstract

Acute lung injury (ALI)/acute respiratory distress syndrome (ARDS) is a severe respiratory condition associated with elevated morbidity and mortality. Understanding their complex pathophysiological mechanisms is crucial for developing new preventive and therapeutic strategies. Recent studies highlight the significant role of inflammation involved in ALI/ARDS, particularly the hyperactivation of the NOD‐like receptor thermal protein domain‐associated protein 3 (NLRP3) inflammasome in macrophages. This activation drives pulmonary inflammation by releasing inflammatory signalling molecules and is linked to metabolic reprogramming, marked by increased glycolysis and reduced oxidative phosphorylation. However, the relationship between NLRP3 inflammasome activation and macrophage glycolytic reprogramming in ALI/ARDS, as well as the molecular mechanisms regulating these processes, remain elusive. This review provides a detailed description of the interactions and potential mechanisms linking NLRP3 inflammasome activation with macrophage glycolytic reprogramming, proposing that glycolytic reprogramming may represent a promising therapeutic target for mitigating inflammatory responses in ALI/ARDS.

**Key points:**

- NLRP3 inflammasome activation is pivotal in mediating the excessive inflammatory response in ALI/ARDS.
- Glycolytic reprogramming regulates NLRP3 inflammasome activation.
- Therapeutic potential of targeting glycolytic reprogramming to inhibit NLRP3 inflammasome activation in ALI/ARDS.

## Graphical abstract

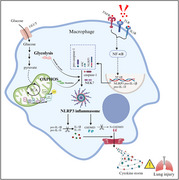

## 1 INTRODUCTION

Acute lung injury (ALI) and acute respiratory distress syndrome (ARDS) are severe clinical conditions characterised by extensive pulmonary inflammation and oedema, elicited by a diverse array of pulmonary and extrapulmonary insults, encompassing pneumonia, systemic infections, ischemia/reperfusion injury, physical trauma, drug‐related toxicity, and mechanical ventilation. These syndromes culminate in acute hypoxemic respiratory failure, with refractory hypoxemia as a cardinal feature.^1^, ^2^, ^3^ Despite advancements in diagnostic and therapeutic strategies, mortality rates for ALI/ARDS remain persistently high at 30%–40%.^4^ The emergence of coronavirus disease‐2019 (COVID‐19) has exacerbated ARDS incidence, with 30%–40% of hospitalised COVID‐19 patients progressing to ARDS, accounting for 70% of COVID‐19‐related mortality, thus placing significant strain on healthcare systems worldwide.^5^, ^6^ However, current therapeutic approaches remain largely palliative, with no definitive curative interventions available. Despite the substantial progress made in elucidating the underlying mechanisms of ALI/ARDS over the past few decades, therapeutic landscape remains dominated by symptomatic relief measures,^7^, ^8^ with a lack of curative treatments.

The pathophysiology underlying ALI/ARDS encompasses intricate and intersecting mechanistic pathways, culminating in an exaggerated inflammatory response within the lungs. This response is characterised by widespread alveolar injury, neutrophilic alveolitis, and hyaline membrane formation.^9^, ^10^, ^11^ In the acute stage of ALI/ARDS, patients often exhibit severe pulmonary inflammatory responses, characterised by a ‘cytokine storm’.^12^ This uncontrolled inflammatory response rapidly generates large amounts of pro‐inflammatory cytokines, such as interleukin‐1β (IL‐1β), interleukin‐6 (IL‐6), tumour necrosis factor‐α (TNF‐α), and interferon‐α. Numerous studies indicate that these mediators primarily originate from inflammasomes.^12^ Inflammasomes are multiprotein complexes that form in reaction to pathogens or cellular stress, activating cysteine aspartic protease 1 (caspase‐1), which then processes and releases pro‐inflammatory cytokines like IL‐1β and interleukin‐18 (IL‐18), thereby driving the inflammatory response and influencing immune system regulation.^13^ Currently, five types of inflammasomes have been identified: NOD‐like receptor thermal protein domain‐associated protein 1 (NLRP1), NOD‐like receptor thermal protein domain‐associated protein 3 (NLRP3), NLR family CARD domain‐containing 4 (NLRC4), ICE protease‐activating factor (IPAF), and Absent in melanoma 2 (AIM2) inflammasomes.^14^ Recently, the NLRP3 inflammasome has garnered broad interest due to its involvement in both the natural and acquired immune systems, which are essential in initiating the inflammatory cascade in ALI/ARDS.

Activated macrophages are key pathological markers of the immune responses and inflammation associated with ALI/ARDS. Alveolar macrophages (AMs) are recognised as the primary drivers of local lung inflammation. Residing on the alveolar epithelial surface within terminal bronchioles and alveolar spaces, they account for 90% to 95% of alveolar cells.^15^ As sentinel cells in the alveolar microenvironment, AMs serve a vital function in maintaining homeostasis, protecting against microbial threats, and regulating inflammatory processes in the lung.^16^ Recent years have confirmed that NLRP3 inflammasome activation in AMs exacerbates pulmonary inflammation. Broadly recognised during ALI/ARDS is the close link between macrophage polarisation and NLRP3 inflammasome activation.^17^, ^18^ The equilibrium between pro‐inflammatory M1 macrophages and anti‐inflammatory M2 macrophages exerts a crucial influence on the repair of lung damage. Interestingly, macrophage polarisation is also accompanied by metabolic pathways alterations.^19^, ^20^ Considerable advancements have been achieved in clarifying the interplay between macrophage glucose metabolism alterations and NLRP3 inflammasome activation in ALI/ARDS.^21^, ^22^

Glucose metabolism primarily involves the pathways of glycolysis and mitochondrial oxidative phosphorylation (OXPHOS). Glycolysis is the anaerobic breakdown of glucose to generate adenosine triphosphate (ATP), whereas OXPHOS is the mitochondrial process of producing ATP through electron transport and oxygen consumption. Under normal conditions, macrophages predominantly rely on OXPHOS for energy.^23^ However, during peak inflammation, they shift to glycolysis in a metabolic adaptation known as glycolytic reprogramming, which meets the increased energy and biosynthetic demands in response to harmful stimuli.^24^, ^25^, ^26^ Recent studies have increasingly demonstrated that glycolytic reprogramming, defined by increased glycolysis and decreased OXPHOS, is associated with NLRP3 inflammasome activation in macrophages over the course of ALI/ARDS.^27^, ^28^ Still, the precise relationship between glycolytic reprogramming and NLRP3 inflammasome activation in macrophages within the period of ALI/ARDS is not fully clarified. It has yet to be fully elucidated which particular metabolites or enzymatic changes directly trigger the activation of NLRP3. Additionally, glycolytic metabolic reprogramming encompasses numerous pathways and feedback mechanisms, and how these pathways influence NLRP3 activity during ALI/ARDS warrants further exploration.

Therefore, elucidating the mechanisms by which macrophage glycolytic reprogramming affects NLRP3 inflammasome activation is critical for advancing the study and treatment of ALI/ARDS. The present review consolidates the current state of knowledge concerning the mechanisms underlying the reprogramming of glucose metabolism that orchestrates macrophage NLRP3 inflammasome activation. Targeting glycolytic reprogramming to inhibit macrophage NLRP3 inflammasome activation is an important strategy to explore anti‐inflammatory therapy of ALI/ARDS.

## 2 TARGETING NLRP3 INFLAMMASOME AGAINST ALI/ARDS

### 2.1 The structure of the NLRP3 inflammasome

NLRP3, a cytosolic pattern recognition receptor that is included in the NOD‐like receptor family, stands as the paramount and most extensively studied member within this class.^29^, ^30^ NLRP3 is a cytosolic protein composed of three domains: a leucine‐rich repeat (LRR) at the C terminal, a central nucleotide‐binding and oligomerisation domain (NACHT) endowed with ATPase activity, and a pyrin domain (PYD) at the N‐terminal. The LRR domains primarily facilitate protein‐protein interactions and contribute to the stability of NLRP3. The NACHT domain, which is capable of nucleotide binding and hydrolysing ATP, engages in ATP‐dependent self‐oligomerisation upon activation, causing interactions with the PYD that recruit apoptosis‐associated speck‐like proteins (ASC).^29^ Exposure to a multitude of noxious stimuli can aberrantly activate NLRP3, prompting its cytoplasmic accumulation and interaction with ASC. ASC then recruits and promotes the maturation of pro‐cysteinyl aspartate specific proteinase‐1 (pro‐caspase‐1) into caspase‐1, which facilitates the transformation of pro‐IL‐1β and pro‐interleukin‐18 (pro‐IL‐18) into the active cytokines IL‐1β and IL‐18. Simultaneously, the catalytically active caspase‐1 dimer breaks the linker that joins the N‐terminal and self‐inhibitory C‐terminal of gasdermin D (GSDMD), releasing the N‐terminal pore‐forming fragment GSDMD‐NT. This N‐terminal fragment oligomerises and forms pores by binding to acidic phospholipids on the plasma membrane,^31^, ^32^ triggering the liberation of IL‐1β and IL‐18.

### 2.2 Activation of the NLRP3 inflammasome

**FIGURE 1:**
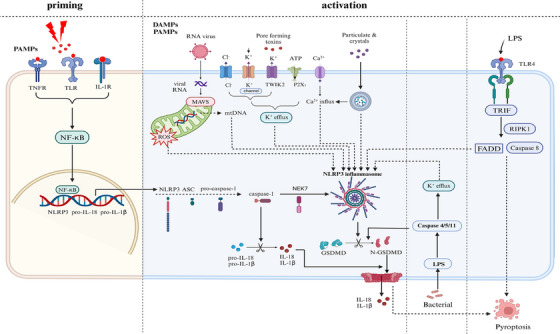
Schematic representation of the possible activation mechanism of NLRP3 inflammasome. The activation of the NLRP3 inflammasome is a two‐step process involving priming and activation. NLRP3 must be primed before activation. Activation of the NLRP3 inflammasome primarily occurs through three mechanisms: classical activation, nonclassical activation, and alternative activation. In the priming phase, deleterious stimuli activate PRRs, such as TLRs, TNFR and IL‐1R, which subsequently triggers NF‐κB signalling, leading to the transcriptional upregulation and increased expression of NLRP3, pro‐caspase‐1, pro‐IL‐1β, and pro‐IL‐18. The classical activation of the NLRP3 inflammasome involves PAMPs, such as toxins and viral RNA, as well as DAMPs, including extracellular ATP, and various particles and crystals. This activation process involves several signalling events, including K^+^ efflux, Ca^2+^ influx, Cl^−^ efflux, lysosomal disruption, production of mtROS, and release of oxidised mtDNA. The nonclassical activation pathway involves phagocytosed bacteria releasing LPS into the cytoplasm, which activates human Caspase‐4/5 or mouse Caspase‐11. Caspase‐11 then cleaves GSDMD, inducing pyroptosis and indirectly activating the NLRP3 inflammasome. This, in turn, leads to the activation of Caspase‐1 and the release of IL‐1β and IL‐18. The alternative activation pathway is induced by TLR4 receptor activation, such as LPS. This pathway activates the TLR4‐TRIF‐RIPK1‐FADD‐Caspase‐8 signalling cascade, which subsequently leads to the activation of the NLRP3 inflammasome. PRRs, pattern recognition receptors; DAMPs, damage‐associated molecular patterns; IL‐1R: interleukin 1 receptor; TNFR: tumour necrosis factor receptor; IL‐18, interleukin‐18; IL‐1β, interleukin‐1β; NLRP3, Nod‐like receptor protein 3; ASC, apoptosis associated with speck‐like proteins; caspase‐1, cysteinyl aspartate specific proteinase‐1; NEK7, NIMA‐related kinase 7; mtDNA, mitochondrial DNA; ROS, reactive oxygen species; GSDMD, gasdermin D; N‐GSDMD, N‐terminal pore‐forming fragment GSDMD; Caspase‐4, cysteinyl aspartate specific proteinase‐4; Caspase‐5, cysteinyl aspartate specific proteinase‐5; Caspase‐11, cysteinyl aspartate specific proteinase‐11; LPS, Lipopolysaccharide; TRIF: TIR domain‐containing adaptor‐inducing interferon‐β; RIPK1: receptor‐interacting protein kinase 1; FADD: Fas‐associated with death domain protein.

NLRP3 inflammasome activation is primarily categorised into three distinct mechanisms: canonical, noncanonical and alternative activation^32^, ^33^, ^34^ (Figure 1). Canonical activation of the NLRP3 inflammasome typically involves two phases: priming and activating signals.^35^ The priming phase is typically driven by Toll‐like receptors (TLRs), IL‐1 receptor and TNF receptor, resulting in the upregulation of NLRP3, pro‐IL‐1β, and pro‐IL‐18 at both transcriptional and translational levels mediated by the nuclear factor kappa B (NF‐κB) signalling pathway. This upregulation furnishes the essential components that enable the subsequent construction and activation of the NLRP3 inflammasome.^36^ Lipopolysaccharide (LPS) serves as a pivotal stimulus for NLRP3 inflammasome activation and is frequently employed for the establishment of ALI/ARDS animal models.^37^ In a mouse model simulating ALI, intratracheal administration of LPS results in elevated IL‐1β concentrations in bronchoalveolar lavage fluid (BALF), knockout of NLRP3 and caspase‐1 attenuates inflammatory cell infiltration within lung tissue.^38^

The activation signal involves NLRP3 recognising pathogen‐associated molecular patterns (PAMPs) or damage‐associated molecular patterns (DAMPs) and assembling into the NLRP3 inflammasome in conjunction with ASC. To date, multiple molecular and cellular events, encompassing extracellular ATP generation, P2X7 receptor‐mediated K^+^ efflux, Ca^2+^ influx, Na^+^ efflux, reactive oxygen species (ROS) release, mitochondrial abnormalities and lysosomal dysfunction, all of which promote NLRP3 activation and assembly^17^, ^36^, ^39^ (Figure 1). Importantly, NLRP3 inflammasome activation relies on NIMA‐related kinase 7 (NEK7),^40^ as its interaction with the LRR domain facilitates the assembly of the NLRP3‐ASC complex, ASC oligomerisation, and the subsequent activation of caspase‐1.^41^ This process typically occurs after mitochondrial ROS (mtROS) induction^42^ and K^+^ efflux.^43^ Research has shown that Entrectinib, a targeted NEK7 inhibitor, exerts significant inhibitory effects in murine models of diseases involved in the NLRP3 inflammasome, such as LPS‐triggered inflammation, monosodium urate‐mediated peritonitis, and type 2 diabetes resulting from a high‐fat diet.^44^

Furthermore, the NLRP3 inflammasome also has noncanonical and alternative activation pathways (Figure 1). Diverging from the canonical activation pathway, the noncanonical activation pathway operates independently of priming signals.^45^ Upon cellular entry, LPS directly binds to caspase‐11/4/5, initiating GSDMD cleavage and leading to pore formation by the GSDMD‐N terminal, which induces K^+^ efflux and activates the NLRP3 inflammasome.^46^, ^47^, ^48^ An alternative activation pathway, observed in human monocytes, represents a novel mechanism for NLRP3 inflammasome activation. Upon toll‐like receptor 4 (TLR4) recognition of LPS, the NLRP3 inflammasome is activated in human monocytes, which subsequently releases IL‐1β and IL‐18 via two distinct pathways. One pathway involves microbial‐induced NLRP3 inflammasome activation via self‐secretion mechanisms; specifically, LPS induces endogenous ATP release from human monocytes, activating the P2X7 receptor and subsequently prompting NLRP3 inflammasome maturation and IL‐1β activation.^49^ The second pathway activates the TLR4‐TIR‐domain‐containing adapter‐inducing interferon‐β (TRIF)‐Receptor‐interacting protein kinase 1 (RIPK1)‐Fas‐associated death domain protein (FADD)‐Caspase‐8 pathway upstream of the NLRP3 inflammasome (Figure 1). Triggered by ATP, perforin toxins, or particulate matter, this mode of activation swiftly releases IL‐1β without typical NLRP3 inflammasome features such as ASC formation, pyroptosis and K^+^ efflux.^50^

### 2.3 Key enzymes and metabolites of glucose metabolic pathways activate the NLRP3 inflammasome

Recently, progress in the area of innate immunity have increasingly shown that NLRP3 not only acts as one of the pattern recognition receptors (PRRs) in the innate immune network but also functions as a sensor for metabolic abnormalities. Various danger signals released during metabolic disruption, such as palmitate,^51^ uric acid^52^ and cholesterol crystals,^53^ have been identified as metabolic‐associated molecular patterns (MAMPs) that can activate NLRP3.^54^ Extensive research has confirmed the critical role of disrupted metabolic pathways in activating the NLRP3 inflammasome.^22^, ^55^, ^56^ For example, high glucose activates NLRP3 through recognition by TLRs, utilising the ATP/P2X7 pathway to activate caspase‐1 and promote NLRP3 inflammasome activation.^57^ During gout and pseudogout episodes, monosodium urate and calcium pyrophosphate crystals trigger the NLRP3 inflammasome and promote IL‐1β release via glucose transporter 1 (GLUT1) receptor engagement on macrophage membranes, exacerbating inflammatory responses.^58^ It has been reported that hexokinase (HK) can act as an PRRs to identify N‐acetylglucosamine (NAC), leading to the dissociation of HK from mitochondria and thereby activating the NLRP3 inflammasome and releasing IL‐1β and IL‐18.^59^ Additionally, the knockout of silent information regulator factor 2‐related enzyme 1 (Sirt1) in cardiac myocytes inhibits pyruvate dehydrogenase (PDH) in a myocardial ischemia‐reperfusion injury model, enhancing ROS production, activating the NLRP3 inflammasome, and leading to myocardial injury.^60^ Similarly, *Salmonella* lacking succinate dehydrogenase (SDH) in the tricarboxylic acid (TCA) cycle elicits the triggering of the NLRP3 inflammasome within infected macrophages, intensifying inflammatory responses.^54^ Macrophage stimulation with LPS results in intracellular succinate accumulation, which activates the hypoxia‐inducible factor‐1α (HIF‐1α)‐mediated NLRP3 inflammasome activation and subsequent release of IL‐1β.^61^ The TCA cycle metabolite itaconate is markedly elevated in inflammatory macrophages and interferes with NLRP3 inflammasome activation through declining the connection between NLRP3 and NEK7.^62^ Besides, itaconate and fumarate derivative pretreatment may inhibit ASC speck formation, thereby suppressing NLRP3 inflammasome assembly.^63^ The glucose fermentation product lactate also contributes to NLRP3 inflammasome activation.^64^ Lactate suppresses TLRs activation in the priming phase via the G‐protein‐coupled receptor 81 (GPR81), inhibiting NF‐κB transcription and NLRP3 inflammasome activation, which alleviating inflammation‐mediated liver and pancreatic damage.^65^ The ketone body β‐hydroxybutyrate is a type of ketone body that functions as an energy source for hepatic tissues in the absence of glucose; however, in the context of urate crystal deposition, ATP, and lipotoxic fatty acids, β‐hydroxybutyrate is reported to suppress NLRP3 inflammasome activation.^66^ Overall, glycolytic reprogramming can influence NLRP3 inflammasome activation across the priming and activation phases.

### 2.4 NLRP3 inflammasome activation exacerbates ALI/ARDS pulmonary inflammation

**FIGURE 2:**
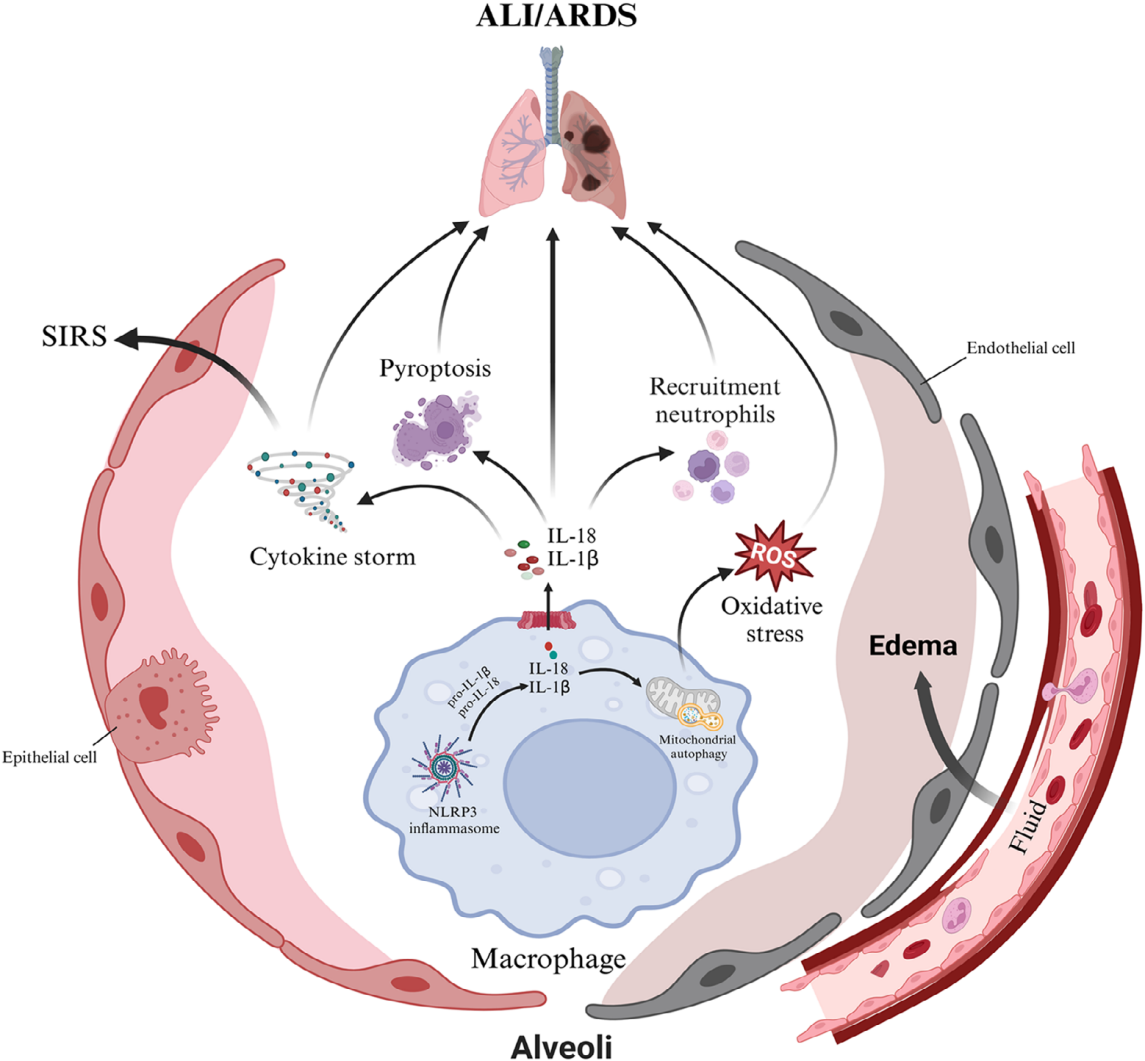
NLRP3 inflammasome activation exacerbates ALI/ARDS pulmonary inflammation. The activation of the NLRP3 inflammasome in AMs releases pro‐inflammatory cytokines IL‐1β and IL‐18, promoting immune cell infiltration, increasing alveolar epithelial permeability, and worsening pulmonary oedema and lung injury. It also induces pyroptosis, releasing danger signals that amplify inflammasome activation. Additionally, it disrupts mitochondrial function, inhibits mitophagy, and increases ROS release, further aggravating lung damage. SIRS, systerm inflammatory response syndrome.

It is now universally recognised that the inflammatory response in ALI/ARDS is intricately linked to the activation of NLRP3 inflammasome (Figure 2). Researches indicate that macrophages from ARDS patients secrete elevated levels of IL‐1β.^67^, ^68^, ^69^ Activation of the NLRP3 inflammasome releases pro‐inflammatory cytokines IL‐1β and IL‐18, further recruiting more immune cells and amplifying local inflammation. For example, notable upregulation of NLRP3 protein expression in lung tissues was observed, correlating with increased expression of IL‐1β and IL‐18 in the LPS‐induced mouse model of ALI. However, NLRP3 knockout reduced the release of these inflammatory mediators, decreased inflammatory cell infiltration in lung tissues, improved alveolar‐capillary permeability, and reduced pulmonary oedema.^38^, ^56^ Similarly, nanochemically modified tetracycline‐3 has emerged as a potent inhibitor of NLRP3 signalling, reducing IL‐1β and IL‐18 levels in both plasma and BALF, ultimately mitigating lung inflammation in ALI mouse models.^70^ Mitochondrial autophagy is notably related to NLRP3 inflammasome activation.^71^ Sestrin2 alleviated ALI by enhancing mitophagy, which protects AMs from pyroptosis and negatively regulates NLRP3 inflammasomes.^72^ Moreover, activation of the NLRP3 inflammasome induces pyroptosis, emitting inflammatory cytokine that damage lung epithelial cells, disrupt alveolar structure, increase capillary permeability, and lead to pulmonary oedema and impaired gas exchange. For instance, metformin inhibits NLRP3‐mediated macrophage pyroptosis through the AMP‐activated protein kinase (AMPK) pathway, thereby mitigating the inflammatory response in sepsis‐related lung injury.^53^ Furthermore, NLRP3 inflammasome activation drives the overproduction of ROS, triggering oxidative stress, increasing permeability, exacerbating lung tissue damage, and impairing gas exchange function.^73^ Additionally, Cui et al. reported that neutrophil extracellular traps trigger the release of excess ROS in macrophages in septic‐related ALI caused by cecal ligation and puncture.^18^ This excess of ROS, in turn, regulates NLRP3 inflammasome activation and post‐translational modification‐mediated necrosis of AMs, thereby accelerating the progression of septic lung injury.^18^ Likewise, cell damage release endogenous danger signals, including high mobility group box 1 (HMGB1),^74^ ATP^75^ and uric acid crystals,^76^ which can activate the NLRP3 inflammasome, establish a positive feedback loop that further intensifies the injury. If uncontrolled, this localised inflammation can lead to a systemic inflammatory response, potentially progressing to ARDS. Overall, overactivation of the NLRP3 inflammasome exacerbates ALI by promoting an excessive inflammatory response, disrupting lung function and leading to potential respiratory failure. While numerous signals that activate NLRP3 have been identified, the specific signals and their recognition mechanisms remain unclear. Additionally, the NLRP3 inflammasome activation encompasses various signalling pathways, necessitating further research into how these signals are coordinated, the specific molecular interactions involved, and how alterations in metabolic pathways influence NLRP3 inflammasome assembly and activation. Consequently, elucidating the mechanisms of NLRP3 inflammasome activation may provide new treatment avenues for inhibiting the inflammatory response in ALI.

## 3 THE INTERPLAY BETWEEN GLYCOLYTIC REPROGRAMMING AND PULMONARY INFLAMMATION IN ALI/ARDS

Even under aerobic conditions, pyruvate can be fermented into lactate without ATP generation, a process commonly termed aerobic glycolysis or the Warburg effect.^77^ During acute inflammatory responses, immune cells primarily rely on glycolysis for energy. Although aerobic glycolysis generates fewer ATP molecules per glucose than mitochondrial OXPHOS, it produces ATP at a much higher rate, supplying the energy needed for critical functions like pathogen phagocytosis, inflammatory mediator synthesis, and cell proliferation.^78^ This rapid ATP generation helps maintain mitochondrial membrane potential and suppresses respiration, limiting ROS accumulation.^79^ The adaptation of glucose metabolism to stress stimuli is termed glycolytic reprogramming. For example, the ‘Warburg effect’ illustrates that cancer cells favour glycolysis for energy needs even in aerobic environments, enabling rapid production of energy and metabolic intermediates necessary for proliferation.^80^ Similarly, activated macrophages undergo glycolytic reprogramming during immune responses, efficiently generating energy and metabolites for functions like inflammatory factor production and cell division.^81^ Notably, glycolytic intermediates regulate energy production, catabolism, and anabolism, and serve as signalling molecules that modulate various protein substrates and key cellular processes, including inflammation, through complex signalling cascades.^81^

### 3.1 The role of glycolytic reprogramming in ALI/ARDS pulmonary inflammation

The human pulmonary alveoli, comprising approximately 90% of total lung capacity, consist of a delicate squamous epithelium enclosed by a capillary‐rich extracellular matrix, constituting the vital interface for the exchange of oxygen and carbon dioxide.^82^ Additionally, the alveolar space is populated by a diverse array of immune cells, including macrophages, neutrophils, dendritic cells, T cells, B cells, and NK cells.^1^, ^13^ Metabolic changes are increasingly recognised as being closely linked to cell fate and functional performance.^78^ Notably, research underscores the pivotal role of immune cell metabolic dysregulation within the alveoli in modulating the pulmonary inflammatory response observed in ALI/ARDS.^83^ Neutrophils, as the initial responders to infection sites and essential players in innate immunity, primarily utilise aerobic glycolysis because of their limited mitochondrial content. Prolonged LPS stimulation suppresses glycolysis in neutrophils, further impairing their chemotactic and phagocytic capabilities during sepsis.^84^ Resting T cells mainly use aerobic oxidative metabolism to process glucose into carbon dioxide, while activated T cells switch to glycolysis or aerobic glycolysis, transforming glucose into lactate to cater to their increased energetic and biosynthetic demands.^85^ Following antigenic stimulation, T cells experience metabolic alterations, characterised by increased lactate levels and enhanced glucose uptake.^86^ Besides, Bart et al. identified that early TLR‐driven glycolytic reprogramming, mediated by the kinases TANK‐binding kinase 1 and IκB Kinase ε, sustains the metabolic requirements of dendritic cell activation.^87^ Laure et al. demonstrated that FP7, a TLR4 antagonist, shields mice from lethal influenza infection by suppressing LPS‐induced cytokine release and glycolytic reprogramming in lung dendritic cells.^88^ The paraquat exposure‐induced ALI model shows that activated macrophages, along with the ensuing release of inflammatory cytokines, are intimately linked to a metabolic shift from OXPHOS to glycolysis.^59^ In summary, metabolic changes not only provide immune cells with necessary energy and metabolic intermediates but also directly affect their function and inflammatory response.

### 3.2 Glycolytic reprogramming of AMs during ALI/ARDS

Pulmonary macrophages, the most abundant cellular population within the alveolar cavity, undergo glycolytic reprogramming, which is closely associated with excessive inflammation in ALI/ARDS.^20^, ^83^ Pulmonary macrophages are primarily composed of AMs and interstitial macrophages, strategically located within the alveoli and pulmonary interstitium, respectively. AMs, lining the alveolar epithelium and representing the most abundant cellular population within the alveolar cavity, function as sentinel cells within the lung, serving as the initial line of defence against lung injury. Their roles are crucial in maintaining pulmonary homeostasis, combating pathogens and regulating pulmonary inflammation.^19^

Like other macrophages, activated AMs are categorised into two states: the M1 phenotype and the M2 phenotype. M1 macrophages possess pro‐inflammatory characteristics, while M2 macrophages exhibit anti‐inflammatory properties.^16^ In response to hypoxic conditions, macrophages undergo a metabolic shift towards glycolysis to ensure survival.^89^, ^90^, ^91^ Macrophages are typically activated to the M1 phenotype by stimuli including LPS,^88^ interferon‐γ (IFN‐γ),^92^ tumour necrosis factor (TNF),^93^ and granulocyte‐macrophage colony‐stimulating factor (GM‐CSF),^94^ promoting the release of pro‐inflammatory cytokines like IL‐6, TNF, IL‐1β, IFN‐β, IL‐12.^95^ IFN‐γ drives the polarisation of M1 macrophage, rapidly activating aerobic glycolysis while decreasing OXPHOS.^96^ Conversely, M2 macrophages are activated by IL‐4,^97^ IL‐13,^98^ IL‐10,^99^ macrophage colony‐stimulating factor,^100^ and IgG,^101^ promoting the expression of anti‐inflammatory cytokines such as IL‐10,^102^ IL‐1 receptor antagonist,^102^ and transforming growth factor‐β.^103^ These cells significantly contribute to the repair of tissue damage during the later stages of inflammation.^96^ Thus, alterations in glucose metabolism are primary determinants of macrophage phenotypic transitions. Upon activation, M1 macrophages undergo a glycolytic reprogramming from OXPHOS to glycolysis to meet increased energy demands and biosynthetic requirements for pro‐inflammatory protein precursors.^104^ In contrast, IL‐4‐induced M2 macrophages exhibit minimal alterations in glucose metabolism, maintaining a stable metabolic state.^105^ Under glucose deprivation, glycolytic in LPS‐stimulated pro‐inflammatory monocytes is significantly inhibited. However, compensatory mechanisms involving the glutamine pathway help maintain cellular function by sustaining TCA cycle flux and releasing IL‐6, TNF, and IL‐1β. This parallels the Warburg effect observed in tumour cells, where activated macrophages exhibit increased activities of glucosekinase and expression, as well as activity of glucose‐6‐phosphate dehydrogenase (G6PD), indicating enhanced glycolysis and pentose phosphate pathway (PPP).^78^

During the early stages of ALI/ARDS, AMs shift to glycolysis for survival in low‐oxygen conditions, and M1 macrophages primarily rely on glycolysis to fulfil their functional roles.^91^, ^106^ This indicates that AMs have undergone glycolytic reprogramming, enabling them to survive under hypoxic conditions and maintain their pro‐inflammatory functions.^91^ Moreover, the expression levels of key glycolysis‐related enzymes, including GLUT1,^107^ hexokinase 2 (HK2),^108^ and lactate dehydrogenase A (LDHA), are elevated in AMs, suggesting glycolytic reprogramming in ALI/ARDS. A mouse ALI model was established via intratracheal injection of LPS, and phloretin inhibited GLUT1‐induced macrophage glycolysis, reducing the inflammatory response during acute lung injury.^107^ The investigation conducted by Rabbani, et al. highlighted the elevated expression of HK2 and G6PD in M1 macrophages, signifying an augmentation in glycolytic flux.^108^ This heightened glycolysis not only provides energy and intermediate metabolites, but also upregulates the rate‐limiting enzymes of the glycolytic pathway, subsequently affecting the secretion of inflammatory factors by M1 macrophages. The accumulation of glycolytic byproducts inhibits the TCA within macrophages, further exacerbating pulmonary inflammatory responses.^109^ In addition, Yang et al. reported a high expression level of HK2 in the lungs, and targeted degradation of HK2 can serve as an anti‐inflammatory treatment in acute lung injury.^110^ Similarly, Eriocitrin suppresses macrophage glycolysis and mitigates pulmonary inflammation by decreasing the expression of HK2 and LDHA.^111^ In conclusion, during ALI/ARDS, AMs undergo glycolytic reprogramming essential for regulating their function and the inflammatory response.

## 4 THE CROSSTALK BETWEEN MACROPHAGE GLYCOLYTIC REPROGRAMMING AND NLRP3 INFLAMMASOME IN ALI/ARDS

Macrophages experience significant metabolic reprogramming triggered by both infectious and sterile stimuli, which is essential for supporting and regulating key innate immune functions, including the activation of the NLRP3 inflammasome.^55^, ^78^, ^81^ Studies have identified glycolytic reprogramming as a crucial factor influencing NLRP3 inflammasome activation in macrophages.^9^, ^28^ Targeting the inhibition of glycolysis‐driven activation of the NLRP3 inflammasome has become a highly promising therapeutic approach for alleviating the pathological changes associated with ALI. Although numerous studies indicate that glycolytic reprogramming promotes NLRP3 inflammasome activation, the precise mechanisms underlying this exacerbation remain largely unexplored and warrant further investigation.^55^, ^81^ In the subsequent sections, we will discuss how changes in OXPHOS and glycolytic pathways influence macrophage NLRP3 inflammasome activation during glycolytic reprogramming.

### 4.1 OXPHOS signals in pulmonary macrophages communication with NLRP3 inflammasome activation

Notably, the electron transport chain (ETC) is crucial for generating and maintaining the mitochondrial membrane potential, that is vital for ATP production under normal conditions.^112^ However, in LPS‐activated macrophages, a decline in mitochondrial membrane potential is observed, facilitating their survival and persistence during immune responses.^79^ LPS stimulation also triggers reverse electron transport (RET), a process where electrons move in reverse through the ETC, inhibiting OXPHOS.^112^, ^113^ RET promotes ROS generation at mitochondrial complex I, stabilising HIF1‐α and ultimately inducing IL‐1β gene expression.^112^ Mitochondrial perturbations have been implicated in inflammasomes activation, which facilitates the processing and release of IL‐1β in macrophages.^14^ Although the precise nature of these mitochondrial perturbations remains unknown, several factors may be involved: the production of mtROS,^114^ mitochondrial dissociation of the glycolytic enzyme HK,^59^ the new mitochondrial DNA synthesis^115^ and altered ETC activity.^116^, ^117^

Importantly, TCA cycle intermediates have roles beyond merely fuelling cells, as recent studies have highlighted their involvement in regulating cell functions, known as the ‘nonmetabolic functions’ of TCA cycle intermediates.^118^ For example, LPS stimulation disrupts TCA cycle activity, influencing the induction of inflammatory genes. This occurs through the upregulation of immune‐responsive gene 1 (IRG1), which leads to the production of itaconate, a metabolite that competitively inhibits SDH, thereby disrupting TCA cycling progression and complex II activity.^119^, ^120^, ^121^ Furthermore, An et al. have reported that quercetin stimulates IRG1‐mediated itaconic production, further inhibiting the SDH/Hif‐1α/NLRP3 signalling pathway.^122^ This inhibition promotes the polarisation of AMs from M1 to M2, mitigating respiratory syncytial virus‐induced lung inflammation.^122^ Mounting evidence highlights the enhanced glycolysis and impaired OXPHOS are hallmarks of M1 macrophages. In these cells, the inhibition of isocitrate dehydrogenase (IDH) leads to citrate accumulation, promoting the synthesis of inflammatory mediators, including nitric oxide (NO) and prostaglandin E2.^114^, ^123^

**FIGURE 3:**
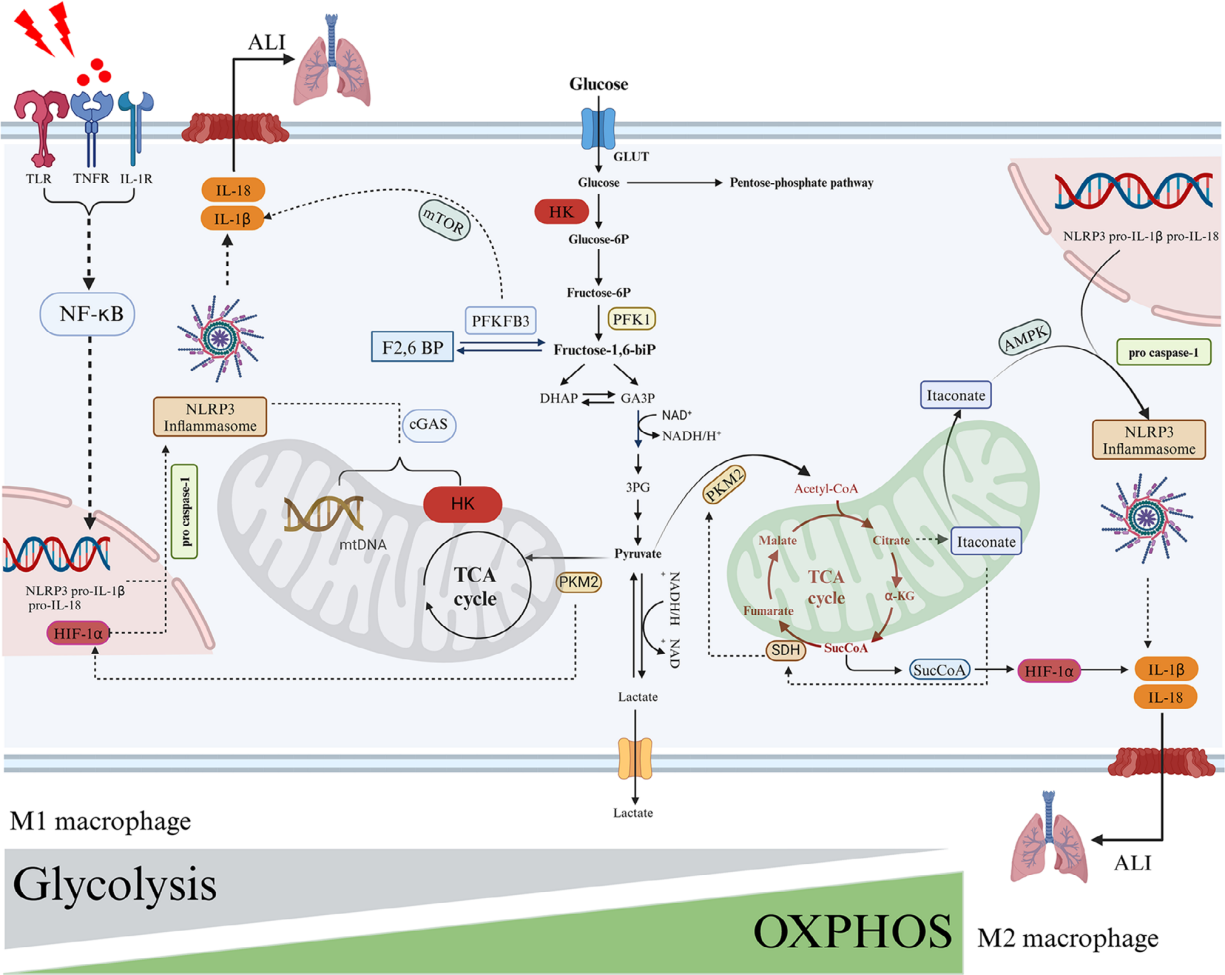
Glycolytic reprogramming in macrophages orchestrates NLRP3 inflammasome activation in ALI/ARDS. Adverse stimuli could activate toll‐like, TNF, and IL‐1 receptors, subsequently leading to the transcriptional activation of pro‐IL‐1β, pro‐IL‐18, NLRP3 and HIF‐1α in an NF‐κB‐dependent manner. Schematic representation of the possible mechanism of glycolysis regulates NLRP3 inflammasome in macrophages. HIF‐1a is a key regulator of glycolysis during hypoxia, upregulate the coding of aerobic glycolysis enzyme at the transcription level in macrophages. Several catalytic enzymes within the glycolytic pathway, including HK, PFKFB3 and PKM, along with accumulated mtDNA, could facilitate the activation of the NLRP3 inflammasome and subsequent secretion of inflammatory cytokines. This process may also be regulated by the mTOR and cGAS pathways. Disrupted mitochondrial homeostasis can result in the accumulation of TCA intermediates, such as succinate and itaconate. Itaconate activates the NLRP3 inflammasome via the AMPK signalling pathway, whereas succinate promotes NLRP3 inflammasome activation through HIF‐1α, leading to the secretion of inflammatory cytokines and exacerbating lung injury. Additionally, glucose metabolic reprogramming is a pivotal factor influencing macrophage phenotype transformation. During ALI, macrophages preferentially engage in aerobic glycolysis, which facilitates their polarisation towards the M1 phenotype. This metabolic shift is associated with a disruption of the TCA cycle and compromised mitochondrial OXPHOS. Conversely, M2 macrophages exhibit a greater reliance on mitochondrial OXPHOS. HIF‐1a, hypoxia‐inducible factor‐1α; PFK, phosphofructokinase; GLUT, glucose transporter; HK, hexokinase; PFKFB3, phosphofructo‐2‐kinase/fructose‐2,6‐bisphosphatase 3; PKM2, pyruvate kinase M2; mTOR, mechanistic target of rapamycin;cGAS, cyclic guanosine monophosphate (cGAMP) synthase; AMPK, AMP‐activated protein kinase; SDH, succinate dehydrogenase; α‐KG, α‐Ketoglutaric acid; SucCoA, succinate CoA.

Additionally, citrate accumulation increases itaconic acid levels, further exacerbating the inhibition of SDH. The resulting succinate can translocate from mitochondria to the cytosol, where it succinylation of lysine residues on proteins, altering the structure and function of pyruvate kinase M2 (PKM2)^124^ (Figure 3). PKM2 subsequently relocates to the nucleus, engages with HIF‐1α, and contributes to the upregulation of IL‐1β expression.^124^ Furthermore, succinate can interact with the G‐protein‐coupled receptor SUCNR1 on the cell membrane, sustaining the pro‐inflammatory phenotype of macrophages.^26^

Fumarate, generated from succinate via SDH activity, is also critical for macrophage polarisation. In LPS‐stimulated macrophages, fumarate promotes M1 polarisation by reducing KDM5 histone demethylase activity and increasing H3K4me3 levels at the TNF and IL‐6 promoters.^125^ Additionally, dimethyl fumarate has been shown to alleviate colitis induced by dextran sulphate sodium through nuclear factor erythroid 2‐related factor 2 (Nrf2) activation coupled with NLRP3 inflammasome activation in colonic macrophages.^126^ In another instance, *Salmonella* deficient in the TCA enzyme aconitase induces NLRP3 inflammasome activation in macrophages during infection, resulting in increased inflammatory responses and diminished virulence.^54^ Notably, the removal of aconitase, isocitrate lyase, or isocitrate dehydrogenase, prompted rapid NLRP3 inflammasome activation in macrophages infected with *Salmonella* enterica serovar Typhimurium.^54^ Similar to DAMPs, extracellular citrate induces NLRP3 inflammasome activation in macrophages, worsening pulmonary inflammation in a mouse model of ALI associated with LPS.^127^

### 4.2 Glycolysis signals in pulmonary macrophages communication with NLRP3 inflammasome activation

In contrast to M1 macrophages, M2 macrophages predominantly rely on OXPHOS and fatty acid oxidation (FAO) for energy production.^128^ It has been demonstrated that inducible nitric oxide synthase (iNOS) inhibits OXPHOS in M1 macrophages, prompting a metabolic and phenotypic reprogramming towards an M2‐like phenotype.^129^ Specifically, NO produced by iNOS competitively binds to cytochrome c oxidase (complex IV) in the ETC, generating reactive nitrogen species,^130^ increasing mitochondrial permeability,^131^ and ultimately reducing the efficiency of OXPHOS. The glycolysis inhibitor 2‐deoxy‐D‐glucose (2‐DG) diminishes the expression of M2‐associated genes such as Arg1, Retnla, Mgl2, and CD206, highlighting the importance of the glycolytic pathway in M2 polarisation.^132^ Metabolic enzymes involved in glycolysis dynamically regulate the inflammatory profile of M1 macrophages.^132^ For example, HK catalyses the conversion of glucose to G6P, which is pivotal for energy generation in glycolysis. The N‐terminal of HK1 contains a mitochondrial binding domain (MBD) that facilitates its mitochondrial association, enabling G6P metabolism via glycolysis.^133^ Nitrosylation by iNOS causes HK1 from mitochondria, redirecting G6P to the pentose phosphate and inhibiting glyceraldehyde‐3‐phosphate dehydrogenase (GAPDH), thereby promoting the release of inflammatory cytokines like IL‐6 and IL‐1β in macrophages.^133^ Moreover, following LPS stimulation, mice lacking the HK1 MBD sequence exhibited an inflammatory cytokine storm.^133^ Similarly, Baik et al. observed that the dissociation of HK from mitochondria facilitates oligomerisation of the voltage‐dependent anion channel, promoting NLRP3 inflammasome assembly and activation in macrophages.^134^ Additionally, synthesis of novel andrographolide Beckmann rearrangement derivatives can suppress the expression of the glycolytic enzyme HK2, which in turn lowers the release of IL‐1β in LPS‐induced RAW264.7 cells.^135^ Zhong et al. reported that inhibition of glycolysis by 2‐DG significantly alleviated pathological lung tissue injury in a mouse model.^136^

Another crucial enzyme regulating glycolysis is phosphofructokinase 1(PFK1), which is modulated by 6‐phosphofructo‐2‐kinase/fructose‐2,6‐bisphosphatase 3 (PFKFB3) (Figure 3). In sepsis‐associated ALI, selective upregulation of PFKFB3 in macrophages enhances the glycolytic pathway, subsequently boosting the release of pro‐inflammatory cytokines. This effect is mediated by Zinc fingers and homeoboxes 2 (Zhx2), which bind to the PFKFB3 promoter.^137^ Conversely, Zhx2 deficiency significantly diminishes the levels of IL‐6 and IL‐1β in M1 macrophages.^137^

Pyruvate kinase, another cornerstone enzyme in glycolysis process, mediates the transformation of phosphoenolpyruvate into pyruvate. Literature has illuminated the role of pyruvate kinase M2 (PKM2)‐related glycolysis activates NLRP3 and AIM2 inflammasomes in macrophages by modulating the phosphorylation of eukaryotic translation initiation factor 2 alpha kinase 2 (EIF2AK2) phosphorylation, resulting in the release of TNF, IL‐1β, and HMGB1.^138^ Moreover, shikonin, a PKM2 inhibitor, can suppress the NLRP3 inflammasome activation, thereby preventing pyroptosis in bone marrow‐derived stem cells (BMSCs) during osteoclastogenesis.^139^ Additionally, ethyl pyruvate may inhibit the release of HMGB1 and preserve mitochondrial integrity, playing a protective role against lethality induced by sepsis and endotoxemia by inhibiting NLRP3 inflammasome activation.^140^

Knockdown of LDHA and MCT‐4 decreases the levels of inflammatory cytokines and inhibits macrophage migration.^106^ Additionally, recent studies suggest that lactate, a metabolite of glycolysis, acts as a novel epigenetic regulator through histone lactylation.^141^ Zhang et al. confirmed that lactate induces the acetylation of histone lysine residues (Kla), driving macrophages toward M2 polarisation.^141^ Similarly, histone lactylation not only directly stimulates gene transcription from chromatin but also induces macrophage polarisation and somatic cell reprogramming. Pan et al. reported that the positive feedback loop of glycolysis/H4K12la/PKM2 leads to dysfunction of microglia, exacerbating the progression of Alzheimer's disease.^142^

Moreover, it was found that the conditional knockout of LDHA in macrophages induced a shift towards M1‐type polarisation, accompanied by suppression of vascular endothelial growth factor (VEGF) expression, impaired vascular repair and enhanced effector CD8+ T cell function.^143^, ^144^ Lactate accumulation enhances the levels of VEGF, Arg1, and other markers of M2‐type macrophages through HIF‐1α signalling.^145^ In a model of sepsis induced by LPS/ATP, activation of NLRP3 inflammasome hinges on heightened glycolysis and increased mitochondrial antiviral signalling protein expression.^146^ Machado et al. revealed that acetate ameliorates NLRP3 inflammasome activation in AMs by targeting the HIF‐1α‐glycolysis axis, thereby reducing IL‐1β production and mitigating pulmonary inflammation triggered by *Streptococcus pneumoniae* infection.^147^ Similarly, Zhong et al. confirmed that activated triggering receptor expressed on myeloid cells‐1 (TREM‐1) promotes HIF‐1α accumulation through the PI3K/AKT/mTOR pathway, leading to enhanced glycolysis in macrophages and activation of the NLRP3 inflammasome in ALI.^148^ Therefore, targeting glycolysis in macrophages emerges as a promising strategy to mitigate NLRP3 inflammasome activation and attenuate lung inflammation. Table 1 summarizes the glycolytic metabolites and enzymes involved in the activation of the NLRP3 inflammasome.

**TABLE 1:** The role of glycolytic metabolites and enzymes in the activation of the NLRP3 inflammasome.

Target	Animal models	Mechanism	Reference
**TCA metabolites**			
PDH	Mice myocardial I/R	Sirt1 inhibits PDH activity, increases ROS and activates the NLRP3 inflammasome	^60^
Citrate	Mice ALI	Activate the NLRP3 inflammasome	^127^
Aconitase	Mouse acute infection	*Salmonella* lacking the TCA enzyme aconitase trigger NLRP3 inflammasome activation	^54^
SDH	Mouse acute infection	*Salmonella* lacking the SDH trigger NLRP3 inflammasome activation	^54^
	Mouse BMDMs	Promote ROS‐mediated GSDMD oligomerisation releasing IL‐1β	^204^
Succinate	Mice sepsis	Activate the HIF‐1α‐mediated NLRP3 inflammasome activation	^61^
Fumarate	Human PBMCs Mouse BMDMs	Inhibit ASC speck formation and suppress NLRP3 inflammasome assembly	^63^
	Murine colitis	Activate Nrf2 and suppress NLRP3 inflammasome activation	^126^
Itaconate	Cryopyrin‐associated periodic syndrome PBMCs Mouse BMDMs	Reduce the NLRP3‐NEK7 interaction to block the activation of the NLRP3 inflammasome.	^62^
	Mice respiratory syncytial virus infection	Inhibit SDH/Hif‐1α/NLRP3 signalling pathway	^122^
	Mouse BMDMs	Suppress IL‐1β secretion and strongly enhance interferon‐β secretion	^122^
	Murine colitis	Activate Nrf2 and suppress NLRP3 inflammasome activation	^126^
**Glycolytic enzymes/metabolites**			
GLUT1	Crystal‐induced inflammation in macrophages	Activate the NLRP3 inflammasome and stimulate IL‐1β release	^58^
HK1	Paraquat‐induced ALI	Inhibit the ERK/MAPK/HK1 pathway	^83^
HK2	Mice ALI Mice primary peritoneal macrophages	Excessive production of IL‐1β	^111^
	Mice BMDMs	Bacterial NAC of HK2 from mitochondria activating the NLRP3 inflammasome	^59^
	RAW264.7 cells	Inhibit HK2 activity and reduce IL‐1β release	^135^
	Human PBMCs	Inhibit the Akt‐mTOR‐STAT3 signalling axis and enhances HK2 expression	^193^
PKM2	Mouse peritoneal macrophages Mouse PBMCs	Promote EIF2AK2 phosphorylation to activate the NLRP3 inflammasome in macrophages	^138^
	Rat cecum ligation and puncture induce sepsis	Downregulate the TLR4/HIF‐1α/PKM2 signalling pathway	^154^
Pyruvate	Peritoneal mouse macrophages Human PBMCs Human THP‐1	Ethyl pyruvate inhibits NLRP3 inflammasome activation via preservation of mitochondrial integrity	^140^
PFKFB3	Mouse sepsis	Zhx2 enhances the glycolytic pathway by binding to the Pfkfb3 promoter, promoting the release of IL‐1	^137^
	Human extravillous trophoblast cell	Metformin inhibits the glycolytic pathway and redox imbalance through the TLR4/NF‐κB/PFKFB3 signalling pathway, suppressing the activation of the NLRP3 inflammasome	^183^
LDHA	Mouse acute pancreatitis Mouse‐derived PAC line 266‐6	Active HIF‐1*α*‐mediated LDHA and NLRP3 signalling pathway	^152^

## 5 POTENTIAL MECHANISMS OF GLYCOLYTIC REPROGRAMMING IN REGULATING NLRP3 ACTIVATION IN PULMONARY MACROPHAGES

Significant advancements have been achieved in elucidating the intricate molecular signals and mechanisms by which macrophage glycolytic reprogramming regulates NLRP3 inflammasome activation. Consequently, a deeper comprehension of the pivotal roles played by key components in glucose metabolic pathways holds the promise of identifying potential therapeutic targets to mitigate excessive pathological inflammation during ALI. In the present review, we consolidate existing insights into the common downstream pathways involved in macrophage glycolytic reprogramming, which ultimately modulate NLRP3 inflammasome activation during ALI.

### 5.1 HIF‐1α pathway

HIF‐1α functions as a pivotal orchestrator of metabolic dynamics, exerting a profound influence on immune responses. Extensive research has elucidated its role as a central regulator in initiating the reconfiguration of glucose metabolism pathways.^149^ Through its N‐terminal activation domain, HIF‐1α forms a heterodimer with HIF‐1β, termed HIF‐1, which subsequently interacts with hypoxia response elements within the genome, triggering a cascade of transcriptional events.^150^ This cascade culminates in the transcriptional activation of crucial glycolytic enzymes, including GLUT‐1, PDH, HK2 and lactate dehydrogenase, which are pivotal for energy production and metabolic adaptation under stress conditions.^151^ Rong et al. reported that blocking xanthine oxidase alleviated pancreatic necrosis in acute pancreatitis by activating HIF‐1α‐regulated LDHA and NLRP3 signalling pathways.^152^ Furthermore, HIF‐1α’s critical involvement in macrophage‐mediated inflammation is underscored by studies demonstrating that its binding proximal to the IL‐1β gene's start site potentiates IL‐1β transcription, a pivotal event in pro‐inflammatory responses.^153^ Suppression HIF‐1α in macrophages significantly attenuates glycolytic flux, causing a decrease in pro‐inflammatory cytokine release by M1 macrophages as well as modulating immune responses.^153^, ^154^ Xijiao Dihuang decoction has been identified as an inhibitor of aerobic glycolysis in macrophages, effectively reducing the levels of inflammatory mediators and cytokines, thereby halting the progression of sepsis.^154^ This therapeutic effect is mediated through the suppression of the TLR4/HIF‐1α/PKM2 signalling pathway, highlighting its potential impact on metabolic reprogramming and inflammation control.^154^ Moreover, Woods et al. have discovered that HIF‐1α induces robust glycolytic reprogramming in AMs, promoting cellular survival during ALI and underscoring its adaptive role in respiratory distress scenarios.^155^ Pan et al. have further established a link between HIF‐1α‐dependent glycolysis and enhanced monocyte inflammatory responses during SARS‐CoV‐2 infection, implicating it in the viral pathogenesis. Additionally, HIF‐1α signalling has been implicated in NLRP3 inflammasome activation, with its inhibition shown to mitigate macrophage NLRP3 inflammasome activation in cerebral ischemia‐reperfusion models, suggesting a broader role in inflammatory pathologies.^156^ In a separate study, Zhong et al. uncovered that activating TREM‐1 amplifies HIF‐1α synthesis in AMs during ALI, which enhanced the glycolysis pathway driving NLRP3 inflammasome activation.^148^ This highlights the intricate interplay between immune signalling pathways and metabolic regulation in the context of lung inflammation.^148^ Targeting HIF‐1α‐related pathways in AMs emerges as a promising strategy to modulate cellular glucose metabolism pathways and thereby regulate inflammatory responses effectively.

### 5.2 AMPK pathway

AMPK, a crucial metabolic regulator essential for maintaining energy balance in response to stress, belongs to the serine/threonine kinase family and is widely expressed across eukaryotic cells.^157^ It functions as a sensitive metabolic sensor, detecting fluctuations in the adenosine monophosphate (AMP) to ATP ratio (AMP:ATP) and subsequently activates catabolic pathways to produce energy. By monitoring AMP and ADP levels relative to ATP, AMPK ensures sufficient energy availability by stimulating catabolic pathways while inhibiting anabolic pathways under stressful conditions.^158^ The AMPK pathway is intricately engaged in regulating inflammation, mitochondrial biogenesis, metabolic reprogramming and responses to infection.^159^, ^160^ As a key sensor of cellular energy status, AMPK activation is not only closely related to catabolic pathways including FAO and OXPHOS, but also involves mTOR signalling and inflammatory cascade responses.^160^ AMPK activation counteracts inflammatory responses by regulating the metabolic shift in innate immune cells, including enhanced early aerobic glycolysis and later oxidative metabolism.^161^ Beyond its role in autophagy modulation, AMPK critically regulates cellular energy homeostasis and bioenergetics by sensing intracellular energy levels in the immune system.^162^

In a mouse model of ARDS, sustained administration of metformin reduces NLRP3 inflammasome activity in macrophages via an AMPK‐dependent pathway, although the exact details remain unclear.^163^ Moreover, metformin inhibits HK2 activity within the peripheral blood mononuclear cells (PBMCs) of individuals with hidradenitis suppurativa via the AMPK‐mTOR pathway, thereby suppressing the NLRP3 inflammasome activation.^164^ Liu et al. demonstrated that Buformin pretreatment upregulates AMPK phosphorylation, enhancing autophagy to degrade the NLRP3 inflammasome and promoting Nrf2 expression to suppress NLRP3 transcription.^165^ Additionally, Tangeretin mitigates sepsis‐induced ALI by inhibiting ROS‐mediated NLRP3 infammasome activation by influencing the PLK1/AMPK/DRP1 signalling axis.^166^ Smiglaside A ameliorates LPS‐induced ALI by driving the transition of AMs to the M2 phenotype by activating the AMPK‐PPARγ pathway.^167^ Piperine suppresses the activation of NLRP3 inflammasome in mouse macrophages (J774A.1) and human proximal renal tubule cells by suppressing extracellular ATP‐induced AMPK phosphorylation.^168^, ^169^ Furthermore, decreased NLRP3 inflammasome activity in the retinas of mice fed a ketogenic diet was linked to the phosphorylation of AMPK.^170^

### 5.3 TLR4 pathway

TLR4, a member of the innate immune PPRs family, is crucial in the pathogenesis of inflammatory diseases, autoimmune disorders and cancers.^171^, ^172^ Extensive studies over the past decades have underscored TLR4's crucial role in regulating glycolipid, closely tied to the pathogenesis of chronic inflammation.^173^, ^174^ TLR4 activates signalling through two separate pathways: myeloid differentiating primary response gene 88 (MyD88)‐dependent and MyD88‐independent pathway.^175^ The MyD88‐dependent pathway, which is paramount in inflammatory diseases, triggers NF‐kB activation and subsequent cytokine production.^176^ Research has highlighted the key involvement of the TLR4/MyD88 signalling pathway in activating the NLRP3 inflammasome,^177^ intertwining TLR4 with MyD88 and NF‐κB, synergistically promote the expression of numerous inflammatory factors.^178^ Fibrinogen‐like protein 2 accelerates the progression of nonalcoholic steatohepatitis by triggering TLR4 signalling to activate the NLRP3 inflammasome in macrophages and contributing to hepatic lipid metabolism disorders.^179^ Zhuang et al. found that the HK2 inhibitor 3‐bromopyruvate (3‐BrPA) exerted therapeutic effects in psoriasis cells and mouse models by downregulating NF‐κB and NLRP3 activation.^180^ The research team led by Eicke Latz discovered that, unlike Warburg metabolism characteristic of advanced macrophage activation, TLR4 signalling quickly increased glucose uptake and boosted both glycolysis and the TCA cycle, resulting in elevated citrate production.^181^ Through MyD88 and TRIF adaptor proteins, TLR4 signalling activated ATP‐citrate lyase, facilitating the use of glucose‐derived acetyl‐Coenzyme A for histones.^181^ This metabolic shift promotes histone acetylation, ultimately driving the enhanced expression of pro‐inflammatory gene sets crucial for a robust inflammatory response. Rafaz et al. demonstrated that in acute liver and pancreatic injury, TLRs activated the inflammasome and stimulate glycolysis to produce lactate, which inhibits TLR‐induced NLRP3 protein activation and IL‐1β secretion via GPR81 and arrestin β‐2.^65^ Additionally, activation of nonclassical STAT3 regulates TLR4‐induced macrophage metabolic reprogramming in the model of LPS‐induced inflammation.^182^ Moreover, Zhang et al. proposed that metformin alleviates pyroptosis triggered by the NLRP3 inflammasome by influencing TLR4/NF‐κB/PFKFB3‐dependent glycolytic reprogramming and redox imbalance.^183^ In a mouse model of LPS‐induced ALI, peficitinib inhibits increased glycolysis and blocks NLRP3 inflammasome activation within macrophages through the TLR4‐mediated JAK3/STAT3 pathway.^184^

### 5.4 mTOR pathway

Rapamycin, a key regulator of cellular metabolism, orchestrates the metabolic shift from OXPHOS to glycolysis in myeloid dendritic cells upon TLR stimulation. This regulation is achieved through the activation of downstream signalling pathways involving HIF‐1α and c‐Myc, which facilitate the transition towards glycolysis.^185^ Moreover, rapamycin directly modulates the expression of enzymes involved in the PPP, further shaping cellular metabolic responses.^186^ In the study by Wu et al. diosgenin was identified as an inhibitor of mTORC2/HIF‐1α signalling, effectively suppressing glycolysis and promoting mTORC2/PPARγ signalling in RAW264.7 cells.^187^ This metabolic shift promotes FAO, which in turn inhibits M1 macrophage polarisation while promoting M2 polarisation. Conversely, mTORC1 enhances glycolysis by upregulating PFKFB3 expression via HIF‐1α, thereby promoting cellular proliferation.^188^ Both LPS and ATP rapidly activate mTOR and glycolysis in microglia.^188^ Direct inhibition of glucose metabolism or mTOR activity significantly reduces the expression of pro‐inflammatory cytokines triggered by LPS, emphasising the important role of mTOR‐driven glycolysis in the pro‐inflammatory response of LPS‐stimulated microglia.^189^ Dexmedetomidine, through its interaction with the α2‐adrenergic receptor/AMPK/mTOR pathway, enhances autophagy and suppresses the activation of NLRP3 inflammasomes, thereby mitigating LPS‐induced acute kidney injury.^190^ Additionally, IL‐21 has been shown to induce pyroptosis of Treg cells in eosinophilic chronic rhinosinusitis through the Akt‐mTOR‐NLRP3‐caspase‐1 axis.^191^ Ip WKE et al. documented that IL‐10 inhibited mTORC1 activation through STAT3 signalling, preserving mitochondrial integrity and attenuating macrophage glycolytic activity, which mitigated NLRP3 inflammasome activation and IL‐1β production, highlighting its role in regulating inflammatory responses.^192^ However, Ahmad et al. demonstrated that all‐trans retinoic acid mitigates the suppression of NLRP3 inflammasome function caused by IL‐10 through inhibiting the Akt‐mTOR‐STAT3 signalling pathway and enhancing HK2 expression.^193^ This shifting alters the metabolism of LPS‐activated human PBMCs toward glycolysis, activating the NLRP3 inflammasome.^193^ In another study, targeting TREM‐1 to inhibit the mTOR/HIF‐1α/glycolytic pathway contributes to the suppression of NLRP3 inflammasome activation in ALI macrophages.^148^

### 5.5 cGAS‐STING axis

Stimulator of interferon gene (STING), also known as mediator of interferon response, transmembrane protein 173, ER‐interferon stimulating, and macrophage stimulating protein, is a pivotal regulator embedded within the endoplasmic reticulum^194^ that activates NF‐κB and interferon regulatory factor 3 pathways, leading to the production of type I interferons.^195^, ^196^ In innate immunity, STING responds to cyclic guanosine monophosphate (cGAMP) synthase (cGAS), influencing diverse pathophysiological processes such as microbial defence, cellular senescence, antitumour immunity, autoimmunity, autophagy, and inflammatory diseases.^197^, ^198^ For example, targeting the cGAS‐STING‐NLRP3 axis in human myeloid cells is proposed to mitigate inflammatory responses during bacterial and viral infections.^199^, ^200^ Zhong et al. have demonstrated that specific inhibition of STING downregulates the activation of the NLRP3 inflammasome, which subsequently lowers the excessive release of IL‐1β and IL‐18 from macrophages, and alleviating liver damage.^201^ In the setting of LPS‐induced lung injury, STING knockout alleviates oxidative stress, inflammation, and cell damage triggered by LPS; however, this protective effect is counteracted by NLRP3 overexpression.^202^ The regulation of STING varies across immune cells. Activated STING induces metabolic reprogramming in macrophages, shifting M2‐polarised macrophages to a pro‐inflammatory M1 phenotype by impairing the TCA cycle, promoting succinate accumulation, inhibiting prolyl hydroxylase activity and enhancing HIF‐1α stability. Furthermore, activation of STING in M1 cells enhances mtROS production, thereby promoting glycolysis while reducing OXPHOS.^195^, ^196^, ^202^ Li et al. discovered that mitochondrial DNA in the cytosol collaborates with c‐Myc to activate and upregulate STING, which subsequently contributes to LPS‐induced ALI by enhancing NLRP3 inflammasome activation and macrophage pyroptosis.^203^

## 6 COMPOUNDS AND DRUGS TARGETING KEY SIGNALLING MOLECULES IN GLYCOLYTIC REPROGRAMMING

In recent years, substances and pharmaceuticals directed at crucial signalling molecules involved in metabolic reprogramming have been increasingly discovered and developed, with some advancing to preclinical and clinical trial stages(Table 2). SDH, also referred to as mitochondrial complex II, facilitates the conversion of succinate to fumarate and simultaneously reduces ubiquinone to ubiquinol, linking the TCA cycle to the ETC. Yang et al. demonstrated that increased SDH activity in liver macrophages during acute liver failure, and its inhibition by dimethyl malonate (DMM), protected mice by reducing NLRP3 inflammasome assembly, GSDMD oligomerisation, and pyroptosis.^204^ HK, the key regulatory enzyme in glycolysis, with several well‐known inhibitors, such as 3‐BrPA, lonidamine, and 2‐DG. In the psoriatic mouse model, 3‐BrPA improves imiquimod‐induced psoriasis‐like dermatitis by downregulating NF‐κB and NLRP3 activation.^180^ Lonidamine inhibits inflammasome activation by directly interacting with ASC, functioning independently of HK2, and exhibits therapeutic efficacy in animal models of various inflammasome‐driven diseases.^205^ Recently, the efficacy of 2‐DG has been evaluated in a phase II clinical trial involving patients with pancreatic cancer.^206^ Lonidamine is gaining recognition as an anticancer agent, utilised both as a standalone treatment and in combination with other therapies. It has finished preclinical studies and is actively enrolled in phase II clinical trials targeting cancer.^207^ DCA exerts its effects by inhibiting the expression of PDK, activating mitochondrial PDH, shifting energy production from glycolysis toward mitochondrial OXPHOS, consuming pyruvate and reducing lactate accumulation.^208^ A single‐arm prospective study assessed the safety of oral DCA in individuals with brain tumours, reporting no significant dose‐limiting toxicities following a minimum of one 4‐week cycle.^209^ A preclinical study demonstrated that shikonin inhibited NLRP3 inflammasome activation, which preventing pyroptosis in BMSCs of osteoclastogenesis.^139^ TEPP‐46, a small‐molecule activator, stabilises the tetrameric structure of the PKM2 subunit, increasing PKM2 kinase activity and inhibiting metabolic reprogramming in pulmonary vascular cells.^210^ The PKFBF3 inhibitor 3‐(3‐pyridinyl)‐1‐(4‐pyridinyl)‐2‐propen‐1‐one (3PO) alleviates sepsis‐induced ALI by reducing leukocyte recruitment through NF‐κB signalling inhibition in endothelial cells.^211^ Inhibitors of LDHA, including FX11 and oxamate, have yielded encouraging results in preclinical studies, although comparable success in clinical trials has not yet been established. FX11, a highly specific inhibitor of LDHA, has been used to deplete LDHA and disrupt mitochondrial functions while depleting the glycolytic reserve in bone marrow‐derived macrophages (BMDMs) of *M. tuberculosis*‐infected C57BL/6J mice.^212^ Oxamate, another inhibitor of LDHA, stimulates intratumoural CD8+ T cell activity by inhibiting tumour cell glycolysis in a mouse model of nonsmall cell lung cancer xenografts when administered alongside an anti‐PD‐1 antibody.^213^ Looking ahead, research on compounds targeting glycolytic reprogramming offers great prospects for the development of innovative therapies for cancer and inflammatory diseases. Advancing these compounds through clinical trials will be crucial in determining their therapeutic potential and expanding their use in personalised medicine.

**TABLE 2:** Pharmaceutical drugs targeting glycolytic reprogramming.

Target	Inhibitor	Clinical trials	Reference
PDH	Dichloroacetate	Phase III trial	^209^
SDH	DMM	Preclinical	^204^
HK2	3‐BrPA	Preclinical	^180^
	Lonidamine	Phase II	^205^
	2‐DG	Phase II	^206^
PKM2	Shikonin	Preclinical	^139^
	TEPP‐46	Preclinical	^210^
PFKFB3	3PO	Preclinical	^211^
LDHA	Oxamate	Preclinical	^213^
	FX11	Preclinical	^212^

## 7 CONCLUSION AND FUTURE PERSPECTIVE

ALI/ARDS is a life‐threatening condition induced by various pulmonary injury factors, involving intricate molecular processes. Notable advancements have occurred regarding NLRP3 inflammasome activation, shedding light on the interplay between macrophage NLRP3 inflammasome activation and lung inflammatory response in recent years. Growing evidence demonstrates the significant roles of glucose metabolic reprogramming in the inflammatory mechanisms connected to ALI/ARDS. Likewise, the NLRP3 inflammasome is postulated to reciprocally influence glucose metabolism, albeit the underlying mechanisms of these interactions, whether direct or mediated via specific signalling cascades or molecules, remain largely elusive. Most studies have focused on LPS/IL‐4‐mediated macrophage inflammation, primarily conducted in vitro. Beyond the known glucose metabolites participating in the modulation of macrophage NLRP3 inflammasome activation, the roles of other metabolites and metabolic enzymes remain unidentified. As such, there is a pressing need for comprehensive studies delving into the reprogramming of macrophage glucose metabolism and its interplay with the NLRP3 inflammasome to deepen our understanding of these processes. This comprehensive review underscores the complex interplay between glucose metabolic reprogramming and unchecked inflammatory responses, specifically its pivotal role in actively facilitating the triggering of the NLRP3 inflammasome in macrophages. Collectively, elucidating these underlying mechanisms represents a crucial step towards developing therapeutic strategies that can reverse, rather than merely attenuate, the progression of ALI/ARDS. Anticipated future research endeavours will delve deeper into the intricacies of metabolic reprogramming during ALI/ARDS, fostering the emergence of innovative therapeutic interventions that specifically target the NLRP3 inflammasome, aiming to mitigate the deleterious consequences of this life‐threatening condition.

In the future, glycolytic reprogramming could become a key target for precisely regulating NLRP3 inflammasome activation to suppress excessive pulmonary inflammation in ALI/ARDS. Advances in targeted anti‐inflammatory therapies will contribute to improving patient outcomes and enhancing the treatment efficacy of ALI/ARDS. The development of drugs targeting glycolytic reprogramming represents a practical application of basic scientific discoveries, with profound implications for human health. Clearly, this will require extensive collaboration among researchers and clinicians, but the outcomes could be transformative.

## AUTHOR CONTRIBUTIONS

Lan Luo wrote the first draft of the manuscript. Xiaoli Zhuang, Lin Fu and Ziyuan Dong drew the figures. Shuyuan Yi and Kan Wang helped search the literature and prepare the manuscript. Yu Jiang and Ju Zhao supervised the work and provided the comments and additional scientific information. Xiaofang Yang and Feilong Hei reviewed and revised the text. All authors read and approved the final manuscript and approved the submitted version.

## CONFLICT OF INTEREST STATEMENT

The authors declare no competing interests.

## ETHICS STATEMENT

Not applicable.

## References

[1] Long Matthew E , Mallampalli Rama K , Horowitz Jeffrey C . Pathogenesis of pneumonia and acute lung injury. Clin Sci (Colch). 2022;136(10):747‐769. 35621124 10.1042/CS20210879 PMC9429452

[2] Gorman EA , O'Kane CM , McAuley DF . Acute respiratory distress syndrome in adults: diagnosis, outcomes, long‐term sequelae, and management. Lancet. 2022;400(10358):1157‐1170. 36070788 10.1016/S0140-6736(22)01439-8

[3] Villar J , Szakmany T , Grasselli G , Camporota L . Redefining ARDS: a paradigm shift. Critical Care. 2023;27(1):416. 37907946 10.1186/s13054-023-04699-w PMC10619227

[4] Bellani G , Laffey JG , Pham T , et al. Epidemiology, patterns of care, and mortality for patients with acute respiratory distress syndrome in intensive care units in 50 countries. JAMA. 2016;315(8):788‐800. 26903337 10.1001/jama.2016.0291

[5] Wu C , Chen X , Cai Y , et al. Risk factors associated with acute respiratory distress syndrome and death in patients with coronavirus disease 2019 pneumonia in Wuhan, China. JAMA Intern Med. 2020;180(7):934‐943. 32167524 10.1001/jamainternmed.2020.0994 PMC7070509

[6] Zhou F , Yu T , Du R , et al. Clinical course and risk factors for mortality of adult inpatients with COVID‐19 in Wuhan, China: a retrospective cohort study. Lancet North Am Ed. 2020;395(10229):1054‐1062. 10.1016/S0140-6736(20)30566-3 PMC7270627 32171076

[7] Matthay MA , Zemans RL , Zimmerman GA , et al. Acute respiratory distress syndrome. Nat Rev Dis Primers. 2019;5(1). 10.1038/s41572-019-0069-0 PMC6709677 30872586

[8] Meyer NJ , Gattinoni L , Calfee CS . Acute respiratory distress syndrome. Lancet North Am Ed. 2021;398(10300):622‐637. 10.1016/S0140-6736(21)00439-6 PMC8248927 34217425

[9] Huppert L , Matthay M , Ware L . Pathogenesis of acute respiratory distress syndrome. Semin Respir Critical Care Med. 2019;40(01):031‐039. 10.1055/s-0039-1683996 PMC7060969 31060086

[10] Hu Q , Zhang S , Yang Y , et al. Extracellular vesicles in the pathogenesis and treatment of acute lung injury. Milit Med Res. 2022;9(1):61. 10.1186/s40779-022-00417-9 PMC9623953 36316787

[11] Bos LDJ , Ware LB . Acute respiratory distress syndrome: causes, pathophysiology, and phenotypes. Lancet. 2022;400(10358):1145‐1156. 36070787 10.1016/S0140-6736(22)01485-4

[12] Wang H , Fan C , Chen X , et al. Pyruvate kinase M2 nuclear translocation regulate ferroptosis‐associated acute lung injury in cytokine storm. Inflammation. 2024;47(5):1667‐1684. 38483700 10.1007/s10753-024-02000-x PMC11549213

[13] Yao J , Sterling K , Wang Z , Zhang Y , Song W . The role of inflammasomes in human diseases and their potential as therapeutic targets. Signal Transduct Targeted Ther. 2024;9(1). 10.1038/s41392-023-01687-y PMC10766654 38177104

[14] Broz P , Dixit VM . Inflammasomes: mechanism of assembly, regulation and signalling. Nat Rev Immunol. 2016;16(7):407‐420. 27291964 10.1038/nri.2016.58

[15] Wang M , Yu F , Chang W , Zhang Y , Zhang L , Li P . Inflammasomes: a rising star on the horizon of COVID‐19 pathophysiology. Front Immunol. 2023;14:1185233. 37251383 10.3389/fimmu.2023.1185233 PMC10213254

[16] Hussell T , Bell TJ . Alveolar macrophages: plasticity in a tissue‐specific context. Nat Rev Immunol. 2014;14(2):81‐93. 24445666 10.1038/nri3600

[17] Su K , Li X‐T , Hong F‐X , Jin M , Xue F‐S . Lidocaine pretreatment attenuates inflammatory response and protects against sepsis‐induced acute lung injury via inhibiting potassium efflux‐dependent NLRP3 activation. Inflamm Res. 2023;72(12):2221‐2235. 37930383 10.1007/s00011-023-01810-3

[18] Cui Y , Yang Y , Tao W , et al. Neutrophil extracellular traps induce alveolar macrophage pyroptosis by regulating NLRP3 deubiquitination, aggravating the development of septic lung injury. J Inflamm Res. 2023;16:861‐877. 36876152 10.2147/JIR.S366436 PMC9983334

[19] Neupane AS , Willson M , Chojnacki AK , et al. Patrolling alveolar macrophages conceal bacteria from the immune system to maintain homeostasis. Cell. 2020;183(1):110‐125 e111. 32888431 10.1016/j.cell.2020.08.020

[20] Dang W , Tao Y , Xu X , Zhao H , Zou L , Li Y . The role of lung macrophages in acute respiratory distress syndrome. Inflamm Res. 2022;71(12):1417‐1432. 36264361 10.1007/s00011-022-01645-4 PMC9582389

[21] Yu Q , Guo M , Zeng W , et al. Interactions between NLRP3 inflammasome and glycolysis in macrophages: New insights into chronic inflammation pathogenesis. Immun, Inflamm Dis. 2021;10(3):e581. 34904398 10.1002/iid3.581 PMC8926505

[22] Hughes MM , O'Neill LAJ . Metabolic regulation of NLRP3. Immunol Rev. 2017;281(1):88‐98. 10.1111/imr.12608 29247992

[23] Zhu J , Thompson CB . Metabolic regulation of cell growth and proliferation. Nat Rev Mol Cell Biol. 2019;20(7):436‐450. 30976106 10.1038/s41580-019-0123-5 PMC6592760

[24] Luo Y , Jiang Q , Zhu Z , et al. Phosphoproteomics and proteomics reveal metabolism as a key node in LPS‐induced acute inflammation in RAW264.7. Inflammation. 2020;43(5):1667‐1679. 32488682 10.1007/s10753-020-01240-x

[25] Luo Y , Qi X , Zhang Z , et al. Inactivation of malic enzyme 1 in endothelial cells alleviates pulmonary hypertension. Circulation. 2024;149(17):1354‐1371. 38314588 10.1161/CIRCULATIONAHA.123.067579

[26] Ye L , Jiang Y , Zhang M . Crosstalk between glucose metabolism, lactate production and immune response modulation. Cytokine Growth Factor Rev. 2022;68:81‐92. 36376165 10.1016/j.cytogfr.2022.11.001

[27] Doughty CA , Bleiman BF , Wagner DJ , et al. Antigen receptor‐mediated changes in glucose metabolism in B lymphocytes: role of phosphatidylinositol 3‐kinase signaling in the glycolytic control of growth. Blood. 2006;107(11):4458‐4465. 16449529 10.1182/blood-2005-12-4788 PMC1895797

[28] Munshi L , Walkey A , Goligher E , Pham T , Uleryk EM , Fan E . Venovenous extracorporeal membrane oxygenation for acute respiratory distress syndrome: a systematic review and meta‐analysis. Lancet Respir Med. 2019;7(2):163‐172. 30642776 10.1016/S2213-2600(18)30452-1

[29] Fu J , Wu H . Structural mechanisms of NLRP3 inflammasome assembly and activation. Annu Rev Immunol. 2023;41(1):301‐316. 36750315 10.1146/annurev-immunol-081022-021207 PMC10159982

[30] Hochheiser IV , Pilsl M , Hagelueken G , et al. Structure of the NLRP3 decamer bound to the cytokine release inhibitor CRID3. Nature. 2022;604(7904):184‐189. 35114687 10.1038/s41586-022-04467-w

[31] Kelley N , Jeltema D , Duan Y , He Y . The NLRP3 inflammasome: an overview of mechanisms of activation and regulation. Int J Mol Sci. 2019;20(13):3328. 31284572 10.3390/ijms20133328 PMC6651423

[32] Huang Y , Xu W , Zhou R . NLRP3 inflammasome activation and cell death. Cellular Mol Immunol. 2021;18(9):2114‐2127. 34321623 10.1038/s41423-021-00740-6 PMC8429580

[33] kodi T , Sankhe R , Gopinathan A , Nandakumar K , Kishore A . New insights on NLRP3 inflammasome: mechanisms of activation, inhibition, and epigenetic regulation. J Neuroimmune Pharmacol. 2024;19(1):7. 38421496 10.1007/s11481-024-10101-5 PMC10904444

[34] Swanson KV , Deng M , Ting JPY . The NLRP3 inflammasome: molecular activation and regulation to therapeutics. Nat Rev Immunol. 2019;19(8):477‐489. 31036962 10.1038/s41577-019-0165-0 PMC7807242

[35] Wang L , Hauenstein AV . The NLRP3 inflammasome: mechanism of action, role in disease and therapies. Mol Aspects Med. 2020;76:100889. 32859386 10.1016/j.mam.2020.100889

[36] Paik S , Kim JK , Silwal P , Sasakawa C , Jo E‐K . An update on the regulatory mechanisms of NLRP3 inflammasome activation. Cellular Mol Immunol. 2021;18(5):1141‐1160. 33850310 10.1038/s41423-021-00670-3 PMC8093260

[37] Zhao W , Ma L , Cai C , Gong X . Caffeine inhibits NLRP3 inflammasome activation by suppressing MAPK/NF‐κB and A2aR signaling in LPS‐induced THP‐1 macrophages. Int J Biol Sci. 2019;15(8):1571‐1581. 31360100 10.7150/ijbs.34211 PMC6643212

[38] Grailer JJ , Canning BA , Kalbitz M , et al. Critical role for the NLRP3 inflammasome during acute lung injury. J Immunol. 2014;192(12):5974‐5983. 24795455 10.4049/jimmunol.1400368 PMC4061751

[39] Zhan X , Li Q , Xu G , Xiao X , Bai Z . The mechanism of NLRP3 inflammasome activation and its pharmacological inhibitors. Front Immunol. 2023;13:1109938. 36741414 10.3389/fimmu.2022.1109938 PMC9889537

[40] Sharif H , Wang L , Wang WL , et al. Structural mechanism for NEK7‐licensed activation of NLRP3 inflammasome. Nature. 2019;570(7761):338‐343. 31189953 10.1038/s41586-019-1295-z PMC6774351

[41] Xiao L , Magupalli VG , Wu H . Cryo‐EM structures of the active NLRP3 inflammasome disc. Nature. 2022;613(7944):595‐600. 36442502 10.1038/s41586-022-05570-8 PMC10091861

[42] Shi H , Wang Y , Li X , et al. NLRP3 activation and mitosis are mutually exclusive events coordinated by NEK7, a new inflammasome component. Nat Immunol. 2015;17(3):250‐258. 26642356 10.1038/ni.3333 PMC4862588

[43] He Y , Zeng MY , Yang D , Motro B , Núñez G . NEK7 is an essential mediator of NLRP3 activation downstream of potassium efflux. Nature. 2016;530(7590):354‐357. 26814970 10.1038/nature16959 PMC4810788

[44] Jin X , Liu D , Zhou X , Luo X , Huang Q , Huang Y . Entrectinib inhibits NLRP3 inflammasome and inflammatory diseases by directly targeting NEK7. Cell Reports Medicine. 2023;4(12):101310. 38118409 10.1016/j.xcrm.2023.101310 PMC10772347

[45] Devant P , Dong Y , Mintseris J , et al. Structural insights into cytokine cleavage by inflammatory caspase‐4. Nature. 2023;624(7991):451‐459. 37993712 10.1038/s41586-023-06751-9 PMC10807405

[46] Liu C , Shen Y , Huang L , Wang J . TLR2/caspase‐5/Panx1 pathway mediates necrosis‐induced NLRP3 inflammasome activation in macrophages during acute kidney injury. Cell Death Discovery. 2022;8(1):232. 35473933 10.1038/s41420-022-01032-2 PMC9042857

[47] Shi J , Zhao Y , Wang K , et al. Cleavage of GSDMD by inflammatory caspases determines pyroptotic cell death. Nature. 2015;526(7575):660‐665. 26375003 10.1038/nature15514

[48] Kayagaki N , Stowe IB , Lee BL , et al. Caspase‐11 cleaves gasdermin D for non‐canonical inflammasome signalling. Nature. 2015;526(7575):666‐671. 26375259 10.1038/nature15541

[49] Wang W , Hu D , Feng Y , et al. Paxillin mediates ATP‐induced activation of P2×7 receptor and NLRP3 inflammasome. BMC Biol. 2020;18(1):182. 33243234 10.1186/s12915-020-00918-w PMC7694937

[50] Gaidt MM , Ebert TS , Chauhan D , et al. Human monocytes engage an alternative inflammasome pathway. Immunity. 2016;44(4):833‐846. 27037191 10.1016/j.immuni.2016.01.012

[51] Guo C , Chi Z , Jiang D , et al. Cholesterol homeostatic regulator SCAP‐SREBP2 integrates NLRP3 inflammasome activation and cholesterol biosynthetic signaling in macrophages. Immunity. 2018;49(5):842‐856 e847. 30366764 10.1016/j.immuni.2018.08.021

[52] Rheinheimer J , de Souza BM , Cardoso NS , Bauer AC , Crispim D . Current role of the NLRP3 inflammasome on obesity and insulin resistance: a systematic review. Metabolism. 2017;74:1‐9. 28764843 10.1016/j.metabol.2017.06.002

[53] Masters SL , Dunne A , Subramanian SL , et al. Activation of the NLRP3 inflammasome by islet amyloid polypeptide provides a mechanism for enhanced IL‐1β in type 2 diabetes. Nat Immunol. 2010;11(10):897‐904. 20835230 10.1038/ni.1935 PMC3103663

[54] Wynosky‐Dolfi MA , Snyder AG , Philip NH , et al. Oxidative metabolism enables Salmonella evasion of the NLRP3 inflammasome. J Exp Med. 2014;211(4):653‐668. 24638169 10.1084/jem.20130627 PMC3978275

[55] Olona A , Leishman S , Anand PK . The NLRP3 inflammasome: regulation by metabolic signals. Trends Immunol. 2022;43(12):978‐989. 36371361 10.1016/j.it.2022.10.003

[56] Sharma BR , Kanneganti T‐D . NLRP3 inflammasome in cancer and metabolic diseases. Nat Immunol. 2021;22(5):550‐559. 33707781 10.1038/s41590-021-00886-5 PMC8132572

[57] Trueblood KE , Mohr S , Dubyak GR . Purinergic regulation of high‐glucose‐induced caspase‐1 activation in the rat retinal Müller cell line rMC‐1. Am J Physiol Cell Physiol. 2011;301(5):C1213‐1223. 21832250 10.1152/ajpcell.00265.2011 PMC3213916

[58] Renaudin F , Orliaguet L , Castelli F , et al. Gout and pseudo‐gout‐related crystals promote GLUT1‐mediated glycolysis that governs NLRP3 and interleukin‐1β activation on macrophages. Ann Rheum Dis. 2020;79(11):1506‐1514. 32699039 10.1136/annrheumdis-2020-217342

[59] Wolf AJ , Reyes CN , Liang W , et al. Hexokinase is an innate immune receptor for the detection of bacterial peptidoglycan. Cell. 2016;166(3):624‐636. 27374331 10.1016/j.cell.2016.05.076 PMC5534359

[60] Han Y , Sun W , Ren D , et al. SIRT1 agonism modulates cardiac NLRP3 inflammasome through pyruvate dehydrogenase during ischemia and reperfusion. Redox Biol. 2020;34:101538. 32325423 10.1016/j.redox.2020.101538 PMC7176991

[61] Tannahill GM , Curtis AM , Adamik J , et al. Succinate is an inflammatory signal that induces IL‐1β through HIF‐1α. Nature. 2013;496(7444):238‐242. 23535595 10.1038/nature11986 PMC4031686

[62] Hooftman A , Angiari S , Hester S , et al. The immunomodulatory metabolite itaconate modifies NLRP3 and inhibits inflammasome activation. Cell Metab. 2020;32(3):468‐478.e467. 32791101 10.1016/j.cmet.2020.07.016 PMC7422798

[63] Hoyle C , Green JP , Allan SM , Brough D , Lemarchand E . Itaconate and fumarate derivatives inhibit priming and activation of the canonical NLRP3 inflammasome in macrophages. Immunology. 2022;165(4):460‐480. 35137954 10.1111/imm.13454 PMC9426622

[64] Lin H‐C , Chen Y‐J , Wei Y‐H , et al. Lactic acid fermentation is required for NLRP3 inflammasome activation. Front Immunol. 2021;12:630380. 33854503 10.3389/fimmu.2021.630380 PMC8039150

[65] Hoque R , Farooq A , Ghani A , Gorelick F , Mehal WZ . Lactate reduces liver and pancreatic injury in Toll‐like receptor– and inflammasome‐mediated inflammation via GPR81‐mediated suppression of innate immunity. Gastroenterology. 2014;146(7):1763‐1774. 24657625 10.1053/j.gastro.2014.03.014 PMC4104305

[66] Youm Y‐H , Nguyen KY , Grant RW , et al. The ketone metabolite β‐hydroxybutyrate blocks NLRP3 inflammasome–mediated inflammatory disease. Nat Med. 2015;21(3):263‐269. 25686106 10.1038/nm.3804 PMC4352123

[67] Peukert K , Fox M , Schulz S , et al. Inhibition of Caspase‐1 with tetracycline ameliorates acute lung injury. Am J Respir Crit Care Med. 2021;204(1):53‐63. 33760701 10.1164/rccm.202005-1916OC PMC8437127

[68] Ramasamy S , Subbian S . Critical determinants of cytokine storm and Type I interferon response in COVID‐19 pathogenesis. Clin Microbiol Rev. 2021;34(3):e00299‐20. 33980688 10.1128/CMR.00299-20 PMC8142516

[69] Boyle AJ , Ferris P , Bradbury I , et al. Baseline plasma IL‐18 may predict simvastatin treatment response in patients with ARDS: a secondary analysis of the HARP‐2 randomised clinical trial. Critical Care. 2022;26(1):164. 35672834 10.1186/s13054-022-04025-w PMC9175337

[70] Meng Q , Wang X , Guo D , et al. Nano‐chemically modified tetracycline‐3 (nCMT‐3) attenuates acute lung injury via blocking sTREM‐1 release and NLRP3 inflammasome activation. Shock. 2022;57(5):749‐758. 35583915 10.1097/SHK.0000000000001927

[71] Kim MJ , Bae SH , Ryu JC , et al. SESN2/sestrin2 suppresses sepsis by inducing mitophagy and inhibiting NLRP3 activation in macrophages. Autophagy. 2016;12(8):1272‐1291. 27337507 10.1080/15548627.2016.1183081 PMC4968237

[72] Wu D , Zhang H , Wu Q , et al. Sestrin 2 protects against LPS‐induced acute lung injury by inducing mitophagy in alveolar macrophages. Life Sci. 2021;267:118941. 33359748 10.1016/j.lfs.2020.118941

[73] Xiang Y , Cai M , Li X , Bao X , Cai D , Li YR . Protective effect of Xiao‐Xu‐Ming decoction‐mediated inhibition of ROS/NLRP3 axis on lipopolysaccharide‐induced acute lung injury in vitro and in vivo. Evid‐Based Complement Altern Med. 2021;2021:1‐10. 10.1155/2021/8257495 PMC8490040 34616481

[74] Zhao Y , Tan S‐W , Huang Z‐Z , et al. NLRP3 inflammasome‐dependent increases in High Mobility Group Box 1 involved in the cognitive dysfunction caused by Tau‐Overexpression. Front Aging Neurosci. 2021;13:721474. 34539383 10.3389/fnagi.2021.721474 PMC8446370

[75] Cicko S , Köhler TC , Ayata CK , et al. Extracellular ATP is a danger signal activating P2×7 receptor in a LPS mediated inflammation (ARDS/ALI). Oncotarget. 2018;9(55):30635‐30648. 30093975 10.18632/oncotarget.25761 PMC6078145

[76] Martinon F , Pétrilli V , Mayor A , Tardivel A , Tschopp J . Gout‐associated uric acid crystals activate the NALP3 inflammasome. Nature. 2006;440(7081):237‐241. 16407889 10.1038/nature04516

[77] Fukushi A , Kim H‐D , Chang Y‐C , Kim C‐H . Revisited metabolic control and reprogramming cancers by means of the Warburg effect in tumor cells. Int J Mol Sci. 2022;23(17):10037. 36077431 10.3390/ijms231710037 PMC9456516

[78] Wang S , Liu R , Yu Q , Dong L , Bi Y , Liu G . Metabolic reprogramming of macrophages during infections and cancer. Cancer Lett. 2019;452:14‐22. 30905817 10.1016/j.canlet.2019.03.015

[79] Zorova LD , Popkov VA , Plotnikov EY , et al. Mitochondrial membrane potential. Anal Biochem. 2018;552:50‐59. 28711444 10.1016/j.ab.2017.07.009 PMC5792320

[80] Fendt S‐M . 100 years of the Warburg effect: a cancer metabolism endeavor. Cell. 2024;187(15):3824‐3828. 39059359 10.1016/j.cell.2024.06.026

[81] Li X , Shen H , Zhang M , et al. Glycolytic reprogramming in macrophages and MSCs during inflammation. Front Immunol. 2023;14:1199751. 37675119 10.3389/fimmu.2023.1199751 PMC10477714

[82] Lim K , Donovan APA , Tang W , et al. Organoid modeling of human fetal lung alveolar development reveals mechanisms of cell fate patterning and neonatal respiratory disease. Cell Stem Cell. 2023;30(1):20‐37 e29. 36493780 10.1016/j.stem.2022.11.013 PMC7618456

[83] Li M , Ren Q , Chen K , et al. Regulation of macrophage polarization and glucose metabolism by the ERK/MAPK‐HK1 signaling pathway in paraquat‐induced acute lung injury. Chem Biol Interact. 2024;397:111062. 38763349 10.1016/j.cbi.2024.111062

[84] Pan T , Sun S , Chen Y , et al. Immune effects of PI3K/Akt/HIF‐1α‐regulated glycolysis in polymorphonuclear neutrophils during sepsis. Critical Care. 2022;26(1):29. 35090526 10.1186/s13054-022-03893-6 PMC8796568

[85] Chen H , Yang T , Zhu L , Zhao Y . Cellular metabolism on T‐cell development and function. Int Rev Immunol. 2014;34(1):19‐33. 24708060 10.3109/08830185.2014.902452

[86] Cammann C , Rath A , Reichl U , et al. Early changes in the metabolic profile of activated CD8+ T cells. BMC Cell Biology. 2016;17(1):28. 27387758 10.1186/s12860-016-0104-x PMC4937576

[87] Everts B , Amiel E , Huang SC‐C , et al. TLR‐driven early glycolytic reprogramming via the kinases TBK1‐IKKɛ supports the anabolic demands of dendritic cell activation. Nat Immunol. 2014;15(4):323‐332. 24562310 10.1038/ni.2833 PMC4358322

[88] Perrin‐Cocon L , Aublin‐Gex A , Sestito SE , et al. TLR4 antagonist FP7 inhibits LPS‐induced cytokine production and glycolytic reprogramming in dendritic cells, and protects mice from lethal influenza infection. Sci Rep. 2017;7(1):40791. 28106157 10.1038/srep40791 PMC5247753

[89] Fan EKY , Fan J . Regulation of alveolar macrophage death in acute lung inflammation. Respir Res. 2018;19(1):50. 29587748 10.1186/s12931-018-0756-5 PMC5872399

[90] Roque W , Romero F . Cellular metabolomics of pulmonary fibrosis, from amino acids to lipids. Am J Physiol Cell Physiol. 2021;320(5):C689‐C695. 33471621 10.1152/ajpcell.00586.2020 PMC8163573

[91] Roiniotis J , Dinh H , Masendycz P , et al. Hypoxia prolongs monocyte/macrophage survival and enhanced glycolysis is associated with their maturation under aerobic conditions. J Immunol. 2009;182(12):7974‐7981. 19494322 10.4049/jimmunol.0804216

[92] Huang Y , Tian C , Li Q , Xu Q . TET1 knockdown inhibits *Porphyromonas gingivalis* LPS/IFN‐γ‐induced M1 macrophage polarization through the NF‐κB pathway in THP‐1 cells. Int J Mol Sci. 2019;20(8):2023. 31022963 10.3390/ijms20082023 PMC6514734

[93] Zhao Y , Xu P , Guo L , et al. Tumor necrosis factor‐α blockade corrects monocyte/macrophage imbalance in primary immune thrombocytopenia. Thromb Haemostasis. 2021;121(06):767‐781. 33469903 10.1055/s-0040-1722186

[94] Jung YJ , Lee Y , Kwon H , et al. Decidual lymphatic endothelial cell‐derived granulocyte‐macrophage colony‐stimulating factor induces M1 macrophage polarization via the NF‐κB pathway in severe pre‐eclampsia. Am J Reprod Immunol. 2023;90(2):e13744. 37491916 10.1111/aji.13744

[95] Orecchioni M , Ghosheh Y , Pramod AB , Ley K . Macrophage polarization: different gene signatures in M1(LPS+) vs. classically and M2(LPS–) vs. alternatively activated macrophages. Front Immunol. 2019;10:1084. 31178859 10.3389/fimmu.2019.01084 PMC6543837

[96] Wang F , Zhang S , Jeon R , et al. Interferon gamma induces reversible metabolic reprogramming of M1 macrophages to sustain cell viability and pro‐inflammatory activity. eBioMedicine. 2018;30:303‐316. 29463472 10.1016/j.ebiom.2018.02.009 PMC5953001

[97] Rao L‐Z , Wang Y , Zhang L , et al. IL‐24 deficiency protects mice against bleomycin‐induced pulmonary fibrosis by repressing IL‐4‐induced M2 program in macrophages. Cell Death Differ. 2020;28(4):1270‐1283. 33144678 10.1038/s41418-020-00650-6 PMC8027679

[98] Lu Y , Han G , Zhang Y , et al. M2 macrophage‐secreted exosomes promote metastasis and increase vascular permeability in hepatocellular carcinoma. Cell Commun Signal. 2023;21(1):299. 37904170 10.1186/s12964-022-00872-w PMC10614338

[99] Lee SJ , Noh SE , Jo DH , Cho CS , Park KS , Kim JH . IL‐10‐induced modulation of macrophage polarization suppresses outer‐blood‐retinal barrier disruption in the streptozotocin‐induced early diabetic retinopathy mouse model. FASEB J. 2024;38(9):e23638. 38713098 10.1096/fj.202400053R

[100] Petrina M , Alothaimeen T , Bouzeineddine NZ , et al. Granulocyte macrophage colony stimulating factor exerts dominant effects over macrophage colony stimulating factor during macrophage differentiation in vitro to induce an inflammatory phenotype. Inflamm Res. 2024;73(2):253‐262. 38158446 10.1007/s00011-023-01834-9

[101] Hu W , Li G , Lin J , et al. M2 macrophage subpopulations in glomeruli are associated with the deposition of IgG subclasses and complements in primary membranous nephropathy. Front Med. 2021;8:657232. 10.3389/fmed.2021.657232 PMC8175664 34095170

[102] Vega‐Galaviz D , Vecchyo‐Tenorio GD , Alcántara‐Suárez R , et al. M2 macrophage immunotherapy abolishes glucose intolerance by increasing IL‐10 expression and AKT activation. Immunotherapy. 2020;12(1):9‐24. 31914828 10.2217/imt-2019-0080

[103] Cai G , Lu Y , Zhong W , et al. Piezo1‐mediated M2 macrophage mechanotransduction enhances bone formation through secretion and activation of transforming growth factor‐β1. Cell Prolif. 2023;56(9):e13440. 36880296 10.1111/cpr.13440 PMC10472522

[104] Müller E , Christopoulos PF , Halder S , et al. Toll‐like receptor ligands and interferon‐γ synergize for induction of antitumor M1 macrophages. Front Immunol. 2017;8:1383. 29123526 10.3389/fimmu.2017.01383 PMC5662546

[105] Anders CB , Lawton TMW , Smith HL , Garret J , Doucette MM , Ammons MCB . Use of integrated metabolomics, transcriptomics, and signal protein profile to characterize the effector function and associated metabotype of polarized macrophage phenotypes. J Leukocyte Biol. 2022;111(3):667‐693. 34374126 10.1002/JLB.6A1120-744R PMC8825884

[106] Kaushik DK , Bhattacharya A , Mirzaei R , et al. Enhanced glycolytic metabolism supports transmigration of brain‐infiltrating macrophages in multiple sclerosis. J Clin Invest. 2019;129(8):3277‐3292. 31112527 10.1172/JCI124012 PMC6668690

[107] Songyang Y , Li W , Li W , Yang J , Song T . The inhibition of GLUT1‐induced glycolysis in macrophage by phloretin participates in the protection during acute lung injury. Int Immunopharmacol. 2022;110:109049. 35853279 10.1016/j.intimp.2022.109049

[108] Rabbani N , Xue M , Thornalley PJ . Hexokinase‐2‐linked glycolytic overload and unscheduled glycolysis – driver of insulin resistance and development of vascular complications of diabetes. Int J Mol Sci. 2022;23(4):2165. 35216280 10.3390/ijms23042165 PMC8877341

[109] Zhang Y , Wang X , Gao Z , et al. Hypoxia‐inducible factor‐1α promotes macrophage functional activities in protecting hypoxia‐tolerant large yellow croaker (*Larimichthys crocea*) against *Aeromonas hydrophila* infection. Front Immunol. 2024;15:1410082. 39156889 10.3389/fimmu.2024.1410082 PMC11327042

[110] Yang J , Dong L , Wang Y , Gong L , Gao H , Xie Y . Targeted degradation of hexokinase 2 for anti‑inflammatory treatment in acute lung injury. Mol Med Rep. 2024;29(5):83. 38516767 10.3892/mmr.2024.13206 PMC10975098

[111] Li D , Yang L , Wang W , et al. Eriocitrin attenuates sepsis‐induced acute lung injury in mice by regulating MKP1/MAPK pathway mediated‐glycolysis. Int Immunopharmacol. 2023;118:110021. 36966548 10.1016/j.intimp.2023.110021

[112] Mills EL , Kelly B , Logan A , et al. Succinate dehydrogenase supports metabolic repurposing of mitochondria to drive inflammatory macrophages. Cell. 2016;167(2):457‐470.e413. 27667687 10.1016/j.cell.2016.08.064 PMC5863951

[113] Jung J , Zeng H , Horng T . Metabolism as a guiding force for immunity. Nat Cell Biol. 2019;21(1):85‐93. 30602764 10.1038/s41556-018-0217-x

[114] Zhou R , Yazdi AS , Menu P , Tschopp J . A role for mitochondria in NLRP3 inflammasome activation. Nature. 2011;469(7329):221‐225. 21124315 10.1038/nature09663

[115] Zhong Z , Liang S , Sanchez‐Lopez E , et al. New mitochondrial DNA synthesis enables NLRP3 inflammasome activation. Nature. 2018;560(7717):198‐203. 30046112 10.1038/s41586-018-0372-z PMC6329306

[116] Sanman LE , Qian Y , Eisele NA , et al. Disruption of glycolytic flux is a signal for inflammasome signaling and pyroptotic cell death. eLife. 2016;5:e13663. 27011353 10.7554/eLife.13663 PMC4846378

[117] Groß CJ , Mishra R , Schneider KS , et al. K(+) efflux‐independent NLRP3 inflammasome activation by small molecules targeting mitochondria. Immunity. 2016;45(4):761‐773. 27692612 10.1016/j.immuni.2016.08.010

[118] Jha AK , Huang SC , Sergushichev A , et al. Network integration of parallel metabolic and transcriptional data reveals metabolic modules that regulate macrophage polarization. Immunity. 2015;42(3):419‐430. 25786174 10.1016/j.immuni.2015.02.005

[119] Lampropoulou V , Sergushichev A , Bambouskova M , et al. Itaconate links inhibition of succinate dehydrogenase with macrophage metabolic remodeling and regulation of inflammation. Cell Metab. 2016;24(1):158‐166. 27374498 10.1016/j.cmet.2016.06.004 PMC5108454

[120] Michelucci A , Cordes T , Ghelfi J , et al. Immune‐responsive gene 1 protein links metabolism to immunity by catalyzing itaconic acid production. Proc Natl Acad Sci. 2013;110(19):7820‐7825. 23610393 10.1073/pnas.1218599110 PMC3651434

[121] Cordes T , Wallace M , Michelucci A , et al. Immunoresponsive gene 1 and itaconate inhibit succinate dehydrogenase to modulate intracellular succinate levels. J Biol Chem. 2016;291(27):14274‐14284. 27189937 10.1074/jbc.M115.685792 PMC4933182

[122] An L , Zhai Q , Tao K , et al. Quercetin induces itaconic acid‐mediated M1/M2 alveolar macrophages polarization in respiratory syncytial virus infection. Phytomedicine. 2024;130:155761. 38797031 10.1016/j.phymed.2024.155761

[123] Alhasawi AA , Thomas SC , Tharmalingam S , Legendre F , Appanna VD . Isocitrate lyase and succinate semialdehyde dehydrogenase mediate the synthesis of α‐ketoglutarate in *Pseudomonas fluorescens* . Front Microbiol. 2019;10:1929. 31507554 10.3389/fmicb.2019.01929 PMC6716453

[124] Wang F , Wang K , Xu W , et al. SIRT5 desuccinylates and activates pyruvate kinase M2 to block macrophage IL‐1β production and to prevent DSS‐induced colitis in mice. Cell Rep. 2017;19(11):2331‐2344. 28614718 10.1016/j.celrep.2017.05.065

[125] Arts RJW , Novakovic B , ter Horst R , et al. Glutaminolysis and fumarate accumulation integrate immunometabolic and epigenetic programs in trained immunity. Cell Metab. 2016;24(6):807‐819. 27866838 10.1016/j.cmet.2016.10.008 PMC5742541

[126] Liu X , Zhou W , Zhang X , et al. Dimethyl fumarate ameliorates dextran sulfate sodium‐induced murine experimental colitis by activating Nrf2 and suppressing NLRP3 inflammasome activation. Biochem Pharmacol. 2016;112:37‐49. 27184504 10.1016/j.bcp.2016.05.002

[127] Duan J‐X , Jiang H‐L , Guan X‐X , et al. Extracellular citrate serves as a DAMP to activate macrophages and promote LPS‐induced lung injury in mice. Int Immunopharmacol. 2021;101:108372. 34810128 10.1016/j.intimp.2021.108372

[128] Pokharel MD , Marciano DP , Fu P , et al. Metabolic reprogramming, oxidative stress, and pulmonary hypertension. Redox Biol. 2023;64:102797. 37392518 10.1016/j.redox.2023.102797 PMC10363484

[129] Liang Y , Yang N , Pan G , Jin B , Wang S , Ji W . Elevated IL‐33 promotes expression of MMP2 and MMP9 via activating STAT3 in alveolar macrophages during LPS‐induced acute lung injury. Cell Mol Biol Lett. 2018;23:52. 30410547 10.1186/s11658-018-0117-x PMC6208075

[130] Santhanam S , Rajamanickam S , Motamarry A , et al. Mitochondrial electron transport chain complex dysfunction in the colonic mucosa in ulcerative colitis. Inflamm Bowel Dis. 2012;18(11):2158‐2168. 22374887 10.1002/ibd.22926

[131] Hardeland R . Melatonin and the electron transport chain. Cell Mol Life Sci. 2017;74(21):3883‐3896. 28785805 10.1007/s00018-017-2615-9 PMC11107625

[132] Zhao Q , Chu Z , Zhu L , et al. 2‐Deoxy‐d‐glucose treatment decreases anti‐inflammatory M2 macrophage polarization in mice with tumor and allergic airway inflammation. Front Immunol. 2017;8:637. 28620389 10.3389/fimmu.2017.00637 PMC5451502

[133] De Jesus A , Keyhani‐Nejad F , Pusec CM , et al. Hexokinase 1 cellular localization regulates the metabolic fate of glucose. Mol Cell. 2022;82(7):1261‐1277.e1269. 35305311 10.1016/j.molcel.2022.02.028 PMC8995391

[134] Baik SH , Ramanujan VK , Becker C , Fett S , Underhill DM , Wolf AJ . Hexokinase dissociation from mitochondria promotes oligomerization of VDAC that facilitates NLRP3 inflammasome assembly and activation. Sci Immunol. 2023;8(84):eade7652. 37327321 10.1126/sciimmunol.ade7652 PMC10360408

[135] Wang W , Wu Y , Yang K , et al. Synthesis of novel andrographolide beckmann rearrangement derivatives and evaluation of their HK2‐related anti‐inflammatory activities. Eur J Med Chem. 2019;173:282‐293. 31009914 10.1016/j.ejmech.2019.04.022

[136] Zhong WJ , Yang HH , Guan XX , et al. Inhibition of glycolysis alleviates lipopolysaccharide‐induced acute lung injury in a mouse model. J Cell Physiol. 2019;234(4):4641‐4654. 30256406 10.1002/jcp.27261

[137] Wang Z , Kong L , Tan S , et al. Zhx2 accelerates sepsis by promoting macrophage glycolysis via Pfkfb3. J Immunol. 2020;204(8):2232‐2241. 32179636 10.4049/jimmunol.1901246

[138] Xie M , Yu Y , Kang R , et al. PKM2‐dependent glycolysis promotes NLRP3 and AIM2 inflammasome activation. Nat Commun. 2016;7(1):13280. 27779186 10.1038/ncomms13280 PMC5093342

[139] Li Z , Wang B , Wang R , et al. Identification of PKM2 as a pyroptosis‐related key gene aggravates senile osteoporosis via the NLRP3/Caspase‐1/GSDMD signaling pathway. Int J Biochem Cell Biol. 2024;169:106537. 38342404 10.1016/j.biocel.2024.106537

[140] Li S , Liang F , Kwan K , et al. Identification of ethyl pyruvate as a NLRP3 inflammasome inhibitor that preserves mitochondrial integrity. Mol Med. 2018;24(1):8. 30134814 10.1186/s10020-018-0006-9 PMC6016887

[141] Zhang Y , Jiang H , Dong M , et al. Macrophage MCT4 inhibition activates reparative genes and protects from atherosclerosis by histone H3 lysine 18 lactylation. Cell Rep. 2024;43(5):114180. 38733581 10.1016/j.celrep.2024.114180

[142] Pan RY , He L , Zhang J , et al. Positive feedback regulation of microglial glucose metabolism by histone H4 lysine 12 lactylation in Alzheimer's disease. Cell Metab. 2022;34(4):634‐648 e636. 35303422 10.1016/j.cmet.2022.02.013

[143] Colegio OR , Chu N‐Q , Szabo AL , et al. Functional polarization of tumour‐associated macrophages by tumour‐derived lactic acid. Nature. 2014;513(7519):559‐563. 25043024 10.1038/nature13490 PMC4301845

[144] Stone SC , Rossetti RAM , Alvarez KLF , et al. Lactate secreted by cervical cancer cells modulates macrophage phenotype. J Leukocyte Biol. 2019;105(5):1041‐1054. 30811636 10.1002/JLB.3A0718-274RR

[145] Ye H , Zhou Q , Zheng S , et al. Tumor‐associated macrophages promote progression and the Warburg effect via CCL18/NF‐kB/VCAM‐1 pathway in pancreatic ductal adenocarcinoma. Cell Death Dis. 2018;9(5):453. 29670110 10.1038/s41419-018-0486-0 PMC5906621

[146] Hsu CG , Li W , Sowden M , Chávez CL , Berk BC . Pnpt1 mediates NLRP3 inflammasome activation by MAVS and metabolic reprogramming in macrophages. Cellular Mol Immunol. 2023;20(2):131‐142. 36596874 10.1038/s41423-022-00962-2 PMC9886977

[147] Machado MG , Patente TA , Rouillé Y , et al. Acetate improves the killing of *Streptococcus pneumoniae* by alveolar macrophages via NLRP3 inflammasome and glycolysis‐HIF‐1α axis. Front Immunol. 2022;13:773261. 35126390 10.3389/fimmu.2022.773261 PMC8810543

[148] Zhong W‐J , Liu T , Yang H‐H , et al. TREM‐1 governs NLRP3 inflammasome activation of macrophages by firing up glycolysis in acute lung injury. Int J Biol Sci. 2023;19(1):242‐257. 36594089 10.7150/ijbs.77304 PMC9760435

[149] Shigeta K , Hasegawa M , Hishiki T , et al. IDH2 stabilizes HIF‐1α‐induced metabolic reprogramming and promotes chemoresistance in urothelial cancer. EMBO J. 2023;42(4):e110620. 36637036 10.15252/embj.2022110620 PMC9929641

[150] Zhao Y , Xing C , Deng Y , Ye C , Peng H . HIF‐1α signaling: essential roles in tumorigenesis and implications in targeted therapies. Genes Diseases. 2024;11(1):234‐251. 37588219 10.1016/j.gendis.2023.02.039 PMC10425810

[151] Corcoran SE , O'Neill LAJ . HIF1α and metabolic reprogramming in inflammation. J Clin Invest. 2016;126(10):3699‐3707. 27571407 10.1172/JCI84431 PMC5096812

[152] Rong J , Han C , Huang Y , et al. Inhibition of xanthine oxidase alleviated pancreatic necrosis via HIF‐1α‐regulated LDHA and NLRP3 signaling pathway in acute pancreatitis. Acta Pharm Sin B. 2024;14(8):3591‐3604. 39220867 10.1016/j.apsb.2024.04.019 PMC11365396

[153] Palsson‐McDermott EM , Curtis AM , Goel G , et al. Pyruvate kinase M2 regulates Hif‐1α activity and IL‐1β induction and is a critical determinant of the warburg effect in LPS‐activated macrophages. Cell Metab. 2015;21(1):65‐80. 25565206 10.1016/j.cmet.2014.12.005 PMC5198835

[154] Lu J , Zhang L , Cheng L , et al. Xijiao Dihuang decoction improves prognosis of sepsis via inhibition of aerobic glycolysis. Biomed Pharmacother. 2020;129:110501. 32768976 10.1016/j.biopha.2020.110501

[155] Woods PS , Kimmig LM , Sun KA , et al. HIF‐1α induces glycolytic reprograming in tissue‐resident alveolar macrophages to promote cell survival during acute lung injury. eLife. 2022;11:e77457. 35822617 10.7554/eLife.77457 PMC9323005

[156] Pan P , Shen M , Yu Z , et al. SARS‐CoV‐2 N protein promotes NLRP3 inflammasome activation to induce hyperinflammation. Nat Commun. 2021;12(1):4664. 34341353 10.1038/s41467-021-25015-6 PMC8329225

[157] Carling D . AMPK signalling in health and disease. Curr Opin Cell Biol. 2017;45:31‐37. 28232179 10.1016/j.ceb.2017.01.005

[158] Trefts E , Shaw RJ . AMPK: restoring metabolic homeostasis over space and time. Mol Cell. 2021;81(18):3677‐3690. 34547233 10.1016/j.molcel.2021.08.015 PMC8549486

[159] Jo E‐K , Silwal P , Yuk J‐M . AMPK‐targeted effector networks in mycobacterial infection. Front Microbiol. 2019;10:520. 30930886 10.3389/fmicb.2019.00520 PMC6429987

[160] Sag D , Carling D , Stout RD , Suttles J . Adenosine 5'‐monophosphate‐activated protein kinase promotes macrophage polarization to an anti‐inflammatory functional phenotype. J Immunol. 2008;181(12):8633‐8641. 19050283 10.4049/jimmunol.181.12.8633 PMC2756051

[161] O'Neill LA , Hardie DG . Metabolism of inflammation limited by AMPK and pseudo‐starvation. Nature. 2013;493(7432):346‐355. 23325217 10.1038/nature11862

[162] Williams NC , O'Neill LAJ . A role for the Krebs cycle intermediate citrate in metabolic reprogramming in innate immunity and inflammation. Front Immunol. 2018;9:141. 29459863 10.3389/fimmu.2018.00141 PMC5807345

[163] Duca FA , Côté CD , Rasmussen BA , et al. Metformin activates a duodenal Ampk–dependent pathway to lower hepatic glucose production in rats. Nat Med. 2015;21(5):506‐511. 25849133 10.1038/nm.3787 PMC6104807

[164] Zhong Y , Jin R , Luo R , et al. Diosgenin targets CaMKK2 to alleviate Type II diabetic nephropathy through improving autophagy, mitophagy and mitochondrial dynamics. Nutrients. 2023;15(16):3554. 37630743 10.3390/nu15163554 PMC10459415

[165] Liu B , Wang Z , He R , et al. Buformin alleviates sepsis‐induced acute lung injury via inhibiting NLRP3‐mediated pyroptosis through an AMPK‐dependent pathway. Clin Sci (Colch). 2022;136(4):273‐289. 35132999 10.1042/CS20211156

[166] Liu Y , Zhang Y , You G , et al. Tangeretin attenuates acute lung injury in septic mice by inhibiting ROS‐mediated NLRP3 inflammasome activation via regulating PLK1/AMPK/DRP1 signaling axis. Inflamm Res. 2024;73(1):47‐63. 38147126 10.1007/s00011-023-01819-8

[167] Wang Y , Xu Y , Zhang P , et al. Smiglaside A ameliorates LPS‐induced acute lung injury by modulating macrophage polarization via AMPK‐PPARγ pathway. Biochem Pharmacol. 2018;156:385‐395. 30195731 10.1016/j.bcp.2018.09.002

[168] Liang Y‐D , Bai W‐J , Li C‐G , et al. Piperine suppresses pyroptosis and interleukin‐1β release upon ATP triggering and bacterial infection. Front Pharmacol. 2016;7:390. 27812336 10.3389/fphar.2016.00390 PMC5071324

[169] Peng X , Yang T , Liu G , Liu H , Peng Y , He L . Piperine ameliorated lupus nephritis by targeting AMPK‐mediated activation of NLRP3 inflammasome. Int Immunopharmacol. 2018;65:448‐457. 30388519 10.1016/j.intimp.2018.10.025

[170] Maruyama K , Nemoto E , Yamada S . Mechanical regulation of macrophage function – cyclic tensile force inhibits NLRP3 inflammasome‐dependent IL‐1β secretion in murine macrophages. Inflamm Regener. 2019;39(1):3. 10.1186/s41232-019-0092-2 PMC6367847 30774738

[171] Coutinho‐Wolino KS , Almeida PP , Mafra D , Stockler‐Pinto MB . Bioactive compounds modulating Toll‐like 4 receptor (TLR4)‐mediated inflammation: pathways involved and future perspectives. Nutr Res. 2022;107:96‐116. 36209684 10.1016/j.nutres.2022.09.001

[172] Vallés PG , Gil Lorenzo AF , Garcia RD , Cacciamani V , Benardon ME , Costantino VV . Toll‐like receptor 4 in acute kidney injury. Int J Mol Sci. 2023;24(2):1415. 36674930 10.3390/ijms24021415 PMC9864062

[173] Shi H , Kokoeva MV , Inouye K , Tzameli I , Yin H , Flier JS . TLR4 links innate immunity and fatty acid–induced insulin resistance. J Clin Invest. 2006;116(11):3015‐3025. 17053832 10.1172/JCI28898 PMC1616196

[174] Rogero M , Calder P . Obesity, inflammation, toll‐like receptor 4 and fatty acids. Nutrients. 2018;10(4):432. 29601492 10.3390/nu10040432 PMC5946217

[175] Owen AM , Luan L , Burelbach KR , et al. MyD88‐dependent signaling drives toll‐like receptor‐induced trained immunity in macrophages. Front Immunol. 2022;13:1044662. 36439136 10.3389/fimmu.2022.1044662 PMC9692127

[176] Ciesielska A , Matyjek M , Kwiatkowska K . TLR4 and CD14 trafficking and its influence on LPS‐induced pro‐inflammatory signaling. Cell Mol Life Sci. 2020;78(4):1233‐1261. 33057840 10.1007/s00018-020-03656-y PMC7904555

[177] Huang L , Li Y , Cheng Z , Lv Z , Luo S , Xia Y . PCSK9 promotes endothelial dysfunction during sepsis via the TLR4/MyD88/NF‐κB and NLRP3 pathways. Inflammation. 2023;46(1):115‐128. 35930089 10.1007/s10753-022-01715-z

[178] Feng X , Zhang H , Hu K , et al. Longdan Xiegan decoction ameliorates vulvovaginal candidiasis by inhibiting the NLRP3 inflammasome via the Toll‐like receptor /MyD88 pathway. J Ethnopharmacol. 2024;318(Pt A):116869. 37390876 10.1016/j.jep.2023.116869

[179] Hu J , Wang H , Li X , et al. Fibrinogen‐like protein 2 aggravates nonalcoholic steatohepatitis via interaction with TLR4, eliciting inflammation in macrophages and inducing hepatic lipid metabolism disorder. Theranostics. 2020;10(21):9702‐9720. 32863955 10.7150/thno.44297 PMC7449923

[180] Zhuang L , Ma W , Jiao J . Inhibition of key glycolytic enzyme hexokinase 2 ameliorates psoriasiform inflammation in vitro and in vivo. Clin Cosmet Investig Dermatol. 2023;16:3229‐3239. 10.2147/CCID.S435624 PMC10642575 37965102

[181] Lauterbach MA , Hanke JE , Serefidou M , et al. Toll‐like receptor signaling rewires macrophage metabolism and promotes histone acetylation via ATP‐citrate lyase. Immunity. 2019;51(6):997‐1011.e1017. 31851905 10.1016/j.immuni.2019.11.009

[182] Balic JJ , Albargy H , Luu K , et al. STAT3 serine phosphorylation is required for TLR4 metabolic reprogramming and IL‐1β expression. Nat Commun. 2020;11(1):3816. 32732870 10.1038/s41467-020-17669-5 PMC7393113

[183] Zhang Y , Liu W , Zhong Y , et al. Metformin corrects glucose metabolism reprogramming and NLRP3 inflammasome‐induced pyroptosis via inhibiting the TLR4/NF‐κB/PFKFB3 signaling in trophoblasts: implication for a potential therapy of preeclampsia. Oxid Med Cell Long. 2021;2021:1‐22. 10.1155/2021/1806344 PMC8601820 34804360

[184] Jiang W , Ren J , Li X , Yang J , Cheng D . Peficitinib alleviated acute lung injury by blocking glycolysis through JAK3/STAT3 pathway. Int Immunopharmacol. 2024;132:111931. 38547769 10.1016/j.intimp.2024.111931

[185] Gordan JD , Thompson CB , Simon MC . HIF and c‐Myc: sibling rivals for control of cancer cell metabolism and proliferation. Cancer Cell. 2007;12(2):108‐113. 17692803 10.1016/j.ccr.2007.07.006 PMC3215289

[186] Ecker J , Liebisch G , Englmaier M , Grandl M , Robenek H , Schmitz G . Induction of fatty acid synthesis is a key requirement for phagocytic differentiation of human monocytes. Proc Natl Acad Sci. 2010;107(17):7817‐7822. 20385828 10.1073/pnas.0912059107 PMC2867858

[187] Wu MM , Wang QM , Huang BY , et al. Dioscin ameliorates murine ulcerative colitis by regulating macrophage polarization. Pharmacol Res. 2021;172:105796. 34343656 10.1016/j.phrs.2021.105796

[188] Feng Y , Wu L . mTOR up‐regulation of PFKFB3 is essential for acute myeloid leukemia cell survival. Biochem Biophys Res Commun. 2017;483(2):897‐903. 28082200 10.1016/j.bbrc.2017.01.031

[189] Hu Y , Mai W , Chen L , et al. mTOR‐mediated metabolic reprogramming shapes distinct microglia functions in response to lipopolysaccharide and ATP. Glia. 2019;68(5):1031‐1045. 31793691 10.1002/glia.23760

[190] Yang T , Feng X , Zhao Y , et al. Dexmedetomidine enhances autophagy via α2‐AR/AMPK/mTOR pathway to inhibit the activation of NLRP3 inflammasome and subsequently alleviates lipopolysaccharide‐induced acute kidney injury. Front Pharmacol. 2020;11:790. 32670056 10.3389/fphar.2020.00790 PMC7326938

[191] Chang L , Wu H , Huang W , et al. IL‐21 induces pyroptosis of Treg cells via Akt‐mTOR‐NLRP3‐caspase 1 axis in eosinophilic chronic rhinosinusitis. J Allergy Clin Immunol. 2023;152(3):641‐655 e614. 37164271 10.1016/j.jaci.2023.04.013

[192] Ip WKE , Hoshi N , Shouval DS , Snapper S , Medzhitov R . Anti‐inflammatory effect of IL‐10 mediated by metabolic reprogramming of macrophages. Science. 2017;356(6337):513‐519. 28473584 10.1126/science.aal3535 PMC6260791

[193] Alatshan A , Kovács GE , Aladdin A , Czimmerer Z , Tar K , Benkő S . All‐trans retinoic acid enhances both the signaling for priming and the glycolysis for activation of NLRP3 inflammasome in human macrophage. Cells. 2020;9(7):1591. 32630207 10.3390/cells9071591 PMC7407903

[194] Szwed A , Kim E , Jacinto E . Regulation and metabolic functions of mTORC1 and mTORC2. Physiol Rev. 2021;101(3):1371‐1426. 33599151 10.1152/physrev.00026.2020 PMC8424549

[195] Sun Z , Hornung V . cGAS‐STING signaling. Curr Biol. 2022;32(13):R730‐R734. 35820380 10.1016/j.cub.2022.05.027

[196] Chen C , Xu P . Cellular functions of cGAS‐STING signaling. Trends Cell Biol. 2023;33(8):630‐648. 36437149 10.1016/j.tcb.2022.11.001

[197] Ahn J , Barber GN . STING signaling and host defense against microbial infection. Exp Mol Med. 2019;51(12):1‐10. 10.1038/s12276-019-0333-0 PMC6906460 31827069

[198] Loo TM , Miyata K , Tanaka Y , Takahashi A . Cellular senescence and senescence‐associated secretory phenotype via the cGAS‐STING signaling pathway in cancer. Cancer Sci. 2019;111(2):304‐311. 31799772 10.1111/cas.14266 PMC7004529

[199] Ning L , Wei W , Wenyang J , Rui X , Qing G . Cytosolic DNA‐STING‐NLRP3 axis is involved in murine acute lung injury induced by lipopolysaccharide. Clin Transl Med. 2020;10(7):e228. 33252860 10.1002/ctm2.228 PMC7668192

[200] Zhong W , Rao Z , Rao J , et al. Aging aggravated liver ischemia and reperfusion injury by promoting STING‐mediated NLRP3 activation in macrophages. Aging Cell. 2020;19(8):e13186. 32666684 10.1111/acel.13186 PMC7431827

[201] Li N , Zhou H , Wu H , et al. STING‐IRF3 contributes to lipopolysaccharide‐induced cardiac dysfunction, inflammation, apoptosis and pyroptosis by activating NLRP3. Redox Biol. 2019;24:101215. 31121492 10.1016/j.redox.2019.101215 PMC6529775

[202] Olson GS , Murray TA , Jahn AN , et al. Type I interferon decreases macrophage energy metabolism during mycobacterial infection. Cell Rep. 2021;35(9):109195. 34077724 10.1016/j.celrep.2021.109195 PMC8244443

[203] Ning L , Wei W , Wenyang J , Rui X , Qing G . Cytosolic DNA‐STING‐NLRP3 axis is involved in murine acute lung injury induced by lipopolysaccharide. Clin Transl Med. 2020;10(7):e228. 33252860 10.1002/ctm2.228 PMC7668192

[204] Yang J , Liang J , Huang C , Wu Z , Lei Y . Hyperactivation of succinate dehydrogenase promotes pyroptosis of macrophage via ROS‐induced GSDMD oligomerization in acute liver failure. Mol Immunol. 2024;169:86‐98. 38552285 10.1016/j.molimm.2024.02.004

[205] Chen C , Zhou Y , Ning X , et al. Directly targeting ASC by lonidamine alleviates inflammasome‐driven diseases. J Neuroinflammation. 2022;19(1):315. 36577999 10.1186/s12974-022-02682-w PMC9798610

[206] Coleman MC , Asbury CR , Daniels D , et al. 2‐deoxy‐D‐glucose causes cytotoxicity, oxidative stress, and radiosensitization in pancreatic cancer. Free Radic Biol Med. 2008;44(3):322‐331. 18215740 10.1016/j.freeradbiomed.2007.08.032

[207] Di Cosimo S , Ferretti G , Papaldo P , Carlini P , Fabi A , Cognetti F . Lonidamine: efficacy and safety in clinical trials for the treatment of solid tumors. Drugs Today (Barc). 2003;39(3):157‐174. 12730701 10.1358/dot.2003.39.3.799451

[208] Tian L , Wu D , Dasgupta A , et al. Epigenetic metabolic reprogramming of right ventricular fibroblasts in pulmonary arterial hypertension: a pyruvate dehydrogenase kinase‐dependent shift in mitochondrial metabolism promotes right ventricular fibrosis. Circ Res. 2020;126(12):1723‐1745. 32216531 10.1161/CIRCRESAHA.120.316443 PMC7274861

[209] Dunbar EM , Coats BS , Shroads AL , et al. Phase 1 trial of dichloroacetate (DCA) in adults with recurrent malignant brain tumors. Invest New Drugs. 2014;32(3):452‐464. 24297161 10.1007/s10637-013-0047-4 PMC4455946

[210] Zhang A , Yu F , Yu W , et al. Pyruvate kinase M2 activation protects against the proliferation and migration of pulmonary artery smooth muscle cells. Cell Tissue Res. 2020;382(3):585‐598. 32719938 10.1007/s00441-020-03245-2

[211] Wang L , Cao Y , Gorshkov B , et al. Ablation of endothelial Pfkfb3 protects mice from acute lung injury in LPS‐induced endotoxemia. Pharmacol Res. 2019;146:104292. 31167111 10.1016/j.phrs.2019.104292 PMC7310404

[212] Krishnamoorthy G , Kaiser P , Abu Abed U , et al. FX11 limits *Mycobacterium tuberculosis* growth and potentiates bactericidal activity of isoniazid through host‐directed activity. Dis Model Mech. 2020;13(3):dmm041954. 32034005 10.1242/dmm.041954 PMC7132771

[213] Zhang C , Zhou L , Zhang M , et al. H3K18 lactylation potentiates immune escape of non‐small cell lung cancer. Cancer Res. 2024;84(21):3589‐3601. 39137401 10.1158/0008-5472.CAN-23-3513

